# Ethics in the operating room: a systematic review

**DOI:** 10.1186/s12910-024-01128-7

**Published:** 2024-11-09

**Authors:** Kari Milch Agledahl, Reidar Pedersen

**Affiliations:** 1 Department of Surgery and Orthopaedics, Finnmark Hospital Trust, Hammerfest, Norway; 2 Department of Clinical Medicine, Faculty of Health Sciences, UiT The Arctic University of Norway, Tromsø, Norway; 3 Centre for Medical Ethics, Institute of Health and Society, University of Oslo, Oslo, Norway

**Keywords:** Medical ethics, Clinical ethics, Surgery, Operating room, Surgical ethics, Systematic review

## Abstract

**Background/Objective:**

The act of surgery involves harming vulnerable patients with the intent that the results will improve their health and, ultimately, help the patients. Such activities will inevitably entail moral decisions, yet the ethics of surgery has only recently developed as a field of medical ethics. Within this field, it is striking how few accounts there are of actions within the operating room. The aim of this systematic review was to investigate how much of the scientific publications on surgical ethics focus on what take place inside the operating room and to explore the ethical issues included in the publications that focus on medical ethics in the operating room.

**Methods:**

We conducted a systematic search of the Medline and Embase databases using a PICO model and the search terms “surgery”, “ethics” and “operating room”. Papers were included if they focused on doctors, entailed activities inside the operating room and contained some ethical analysis. Thematic synthesis was used for data extraction and analysis.

**Findings:**

Fewer than 2% of the scientific publications on surgical ethics included activities inside the operating room. A total of 108 studies were included in the full-text analysis and reported according to the RESERVE guidelines. Eight content areas covered 2/3 of the included papers: DNR orders in the OR, overlapping surgery, donation of organs, broadcasting live surgery, video recordings in the OR, communication/teamwork, implementing new surgical technology, and denying blood to Jehovah’s Witness.

**Discussion/Conclusions:**

This systematic review indicates that only a small fraction of scientific publications on the ethics of surgery focus on issues inside the operating room, accentuating the need for further research to close this gap. The ethical issues that repeatedly arose in the included papers included the meaning of patient autonomy inside the operating room, the consequences of technological advances in surgery, the balancing of legitimate interests, the dehumanising potential of the OR, and the strong notion of surgeon responsibility.

**Supplementary Information:**

The online version contains supplementary material available at 10.1186/s12910-024-01128-7.

## Background

Surgery is much more advanced than performing a set procedure, and although technical in nature involves improvisation and on-the-spot decisions [1]. Consequences of surgical complications may be vast, threatening a limb, an organ or even the patient’s life, and surgical decisions thus have major ethical significance. Indeed, a defining feature of surgery is the harm done to the patient even from the first incision. Hopefully, accomplishments of the surgery will exceed the evils of an operation wound, but the ethical proverb often ascribed to Hippocrates, primum non nocere, does not meaningfully apply to surgeons, as they inevitably harm as profession. Assuming personal responsibility for one’s actions and omissions is considered by many surgeons to be a crucial part of surgical training [2].

Another defining ethical feature of surgery is the extreme vulnerability of the patient. Often unconscious, patients literally place their lives in the hands of the surgical team, dependent upon them being kept alive while in the operating room (OR). Unaware of what is being done to their bodies, patients have no choice but to trust their surgeons. Exploitations of this trust have been disclosed; for instance, when unknowingly and without consent, patients have been used for medical students’ gynaecology training [3]. Power imbalance and patient vulnerability in the OR are important ethical issues.

On this backdrop, it is surprising that surgery is scarcely addressed in the field of medical ethics. The most influential book in Western medical ethics, Principles of Biomedical Ethics, only mentions surgery in relation to informed consent [4]. There are partly historical reasons for this omission. While the Hippocratic oath is referred to as the origin of medical ethics, the oath specifically prohibited doctors from performing surgery [5]. Indeed, surgeons were not part of the college of physicians until the 19th century [6]. More recent developments in medical ethics have also overlooked surgery. In line with societal movements to secure individual rights, medical ethics has centred on patient communication, shared decision-making, and clinical ethics committees. Clinical ethics consultations foster an ethics of moral reflection and argumentation [7], which may have made ethicists blind to surgery as a morally relevant endeavour. Ethical issues have been far more prevalent in the medical literature than in the surgical literature [8, 9]. However, within the field of surgery, there has been a growing effort to describe ethical challenges, and the field of surgery ethics has expanded since 2000, especially in American journals [10]. The effort has demonstrated how surgeons are not mere technicians but moral agents [11]. Some papers have insisted that surgery differs from general medicine in ways that call for a distinct surgical ethics [12]. The topics of concurrent surgery, palliative surgery, standards of excellence and surgical innovation indicate that surgical work addresses challenges not correspondingly found in nonsurgical medicine and thus warrants specific ethical reflection [13].

Still, while surgical ethics publications raise expectations for a more relevant and clinically oriented ethics, descriptions of surgical actions within the OR are rare. Attention is often limited to preoperative considerations, such as patient information and consent, or postoperative care, including complications [14, 15]. The activities within the OR are rarely discussed. There may be good reasons for this, as operating theatres are not readily accessible to ethicists and philosophers, but there is also reason to believe that many actions of moral consequence are conducted within that constricted area. Since the OR is the surgeons’ primary workplace, these missing descriptions in the surgical ethics scientific literature are remarkable. We need to explore what these publications cover to determine what might be deliberately or unconsciously left out. With this study, we sought to investigate scientific publications on surgical ethics that focus on what take place *inside* the operating room.

### Research question

How much of the scientific publications on surgical ethics focus on what take place inside the operating room? Which ethical issues are covered by the publications that focus on ethics in the operating room?

## Methods

### Search strategy and eligibility criteria

We conducted a systematic literature search 9th February 2023 using the eight steps described by Droste et al. [16]. The research question was translated into a population, intervention, comparison, and outcome (PICO) model, as shown in Table 1.

**Table 1:** Population, intervention, comparison, and outcome (PICO)

Population	Surgeons
Intervention	Actions and decisions taken inside the operating room (OR)
Comparison	Any or none
Outcome	Ethical aspects emerging from actions and decisions inside the OR

With support from librarians, we developed a search strategy based on three main terms: surgery, ethics, and operating room. We used the corresponding subject headings/MeSH terms as well as free text queries. We explored relevant subject headings/MeSH terms for each of the three main search terms, and synonyms were added to the free text queries. The Boolean operator “OR” was used between synonyms inside each main term, and the operator “AND” was used between the main terms to find the publications that contained all three concepts. Ethical discourse is represented in different types of writings, not only scientific articles; thus, we decided to include smaller texts, such as narratives, comments, and editorials. Conference abstracts, which are more preliminary and incomplete work, as well as papers written in languages other than English or Scandinavian languages, were excluded. The search was conducted in the Medline and Embase databases; see Additional File 1 for the full search string. The resulting articles were exported to Covidence, and duplicates were removed both automatically and manually.

### Selection process

All 1113 papers were screened by title/abstract in Covidence by KMA. Papers were included if they concerned issues that take place inside the OR and had an explicit focus on ethics. The planning of operations or obtaining informed consent was thus excluded, whereas the actions, decisions, and behaviour of the surgeon or OR team inside the OR were included. Additionally, papers had to elaborate on ethical issues in some detail, and only mentioning the presence of ethical issues was not sufficient to be included. Studies that only referred to research ethics were excluded. Both qualitative, quantitative, and nonempirical articles were included. When there was doubt about the article’s focus, some texts were read in full, and if still in doubt, they were discussed with RP. This resulted in 231 articles that were screened in full text by KMA and RP independently. For an overview of the reasons for exclusion, see Table 2. Disagreements or doubts were discussed and resolved by the two authors collectively in separate meetings. A snowball search and hand search resulted in eight additional papers.

**Table 2:** Exclusion reasons for full text screening

	*n*=131
Not explicit focus on ethics	92
Research ethics. Ethics only mentioned in relation to research ethics aspects, e.g. approval from an ethics committee or collecting informed consent prior to research or surgery	1
Actions before or after the OR is the only focus of the article	29
Nurses, OR technicians, etc. The sole focus is on other health care personnel than doctors.	3
Educational environment, OR without patients for training purposes	1
Foreign language, other than English, Norwegian, Danish, or Swedish	4
Full text article not found	1

### Data extraction, coding, and synthesis

Quality appraisal of the papers was a challenging issue, as our search included all genres of publications, including comments and narratives. There is no valid method for measuring a paper’s quality across such diverse publications. Additionally, we aimed to identify ethical aspects of the topics emerging from within the operating room, and even a minor text or a paper of lesser scientific quality could potentially be of interest to the study end. Thus, we did not exclude articles based on quality, but some articles that had a clearer focus on ethics and addressed our research question in more detail were marked as “substantial” and given more weight in the analysis. For each article, passages that discussed ethical issues taking place inside the OR were marked, including all sections of the article. The passages were condensed or converted to keywords that were collected in an extraction sheet, where all articles were represented (see Additional File 2). Data extraction was conducted by KMA.

All articles were examined to identify ethical aspects of actions and decisions within the OR. Some activities in the OR were repeatedly discussed, and we grouped the publications according to these frequently appearing content areas [17]. The extracted keywords and excerpts from the articles within each content area were analysed for similarities and differences and translated to common concepts [18]. We regularly returned to the original texts to ensure proper understanding of the extracts. From this comparative hermeneutic analysis, analytical themes emerged across articles, denoting similar ethical arguments or viewpoints. The coding and analysis were conducted by KMA in consecutive discussions with RP.

## Results

**Fig. 1:**
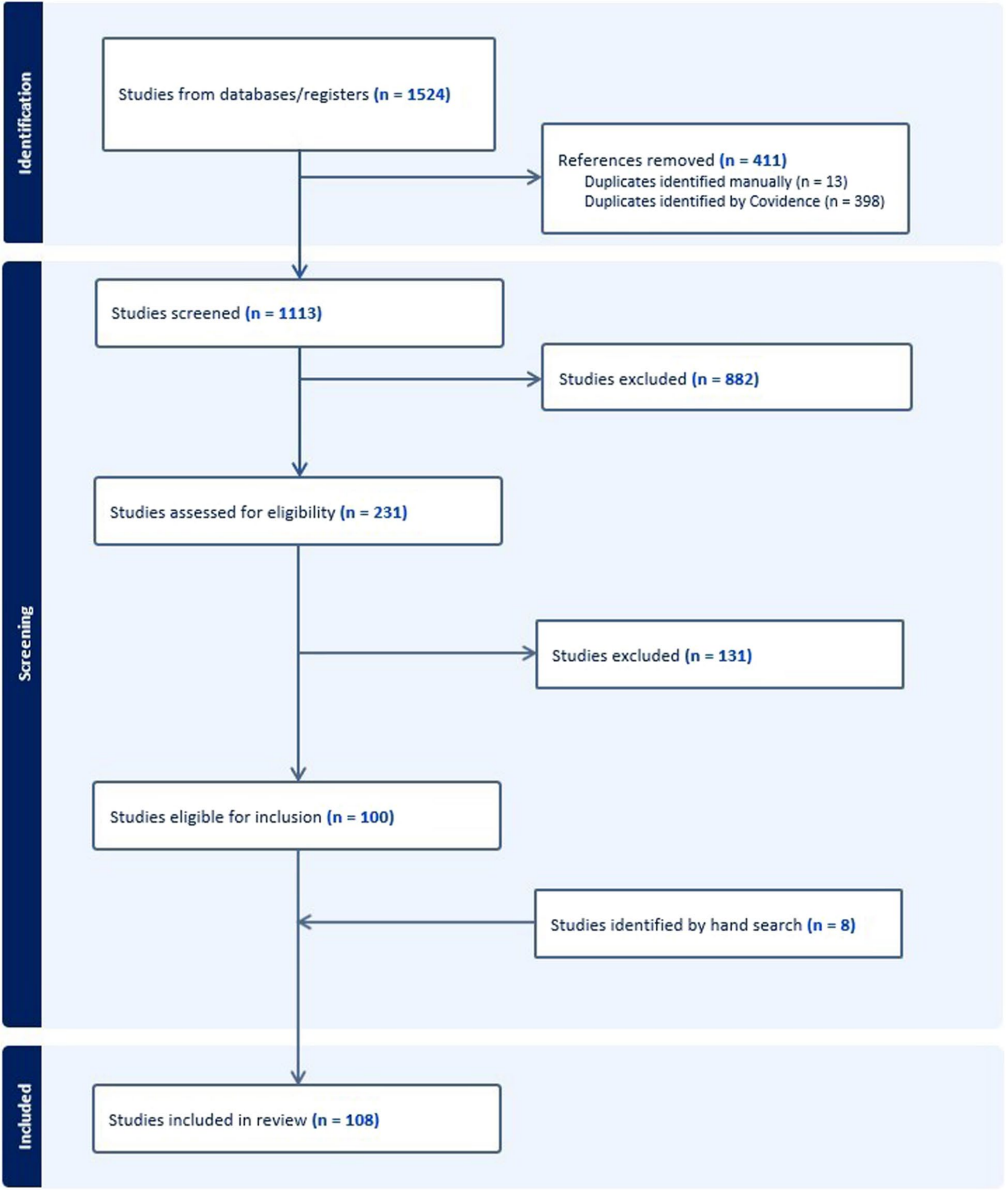
PRISMA flow diagram

Using the two main search terms “surgery” and “ethics” in combination (with the Boolean operator AND), we identified over 40 000 hits in Medline and 60 000 hits in Embase. When we combined this search with “operating room” as the last main search term, the number of hits was reduced to 504 and 1020, respectively; see the Additional File 3 for details. The first search thus resulted in 1524 papers. After removal of duplications, 1113 papers were screened by title and abstract. The resulting 231 papers were read in full text, and 100 papers were found to be eligible. Eight more papers were identified by manual searching; thus, 108 papers were ultimately included in the review (Fig. 1).

The 108 included articles originated from 16 different countries, as identified by the study setting or else the first author. 77 of the articles originated from the United States, 7 from Canada and 6 from the United Kingdom. 11 articles were from other European countries (Belgium, Germany, Greece, Netherlands, France, Sweden, and Norway), 6 were from Asian countries (India, Pakistan, Iran, Japan, and South Korea), and 1 article was from South America (Chile). 51 of the included papers were scientific publications, of which 11 were empirical articles. 11 of the included papers were case discussions, and 10 were guidelines. 26 of the papers, often comments or responses, were less than 2 pages long; see Additional File 2 for details.

We grouped the publications according to the following frequently appearing content areas, which denoted different ethical challenges: DNR orders in the OR, overlapping surgery, donation of organs, broadcasting live surgery, video recordings in the OR, communication/teamwork, implementing new surgical technology, and denying blood to Jehovah’s Witness. These eight areas covered 2/3 of the included papers and are presented below. A summary of the remaining papers is given under the heading “other OR challenges”. In the following, we present each content area, together with the reasons given for it to constitute a challenge inside the OR.

### DNR orders in the OR

The issue of Do-Not-Resuscitate (DNR) orders in the OR is the most covered topic in the publications reviewed. “DNR orders” refer to the medical decision that a patient is not to receive cardiopulmonary resuscitation in the event of cardiac arrest. Patients may have a right to authorise a DNR order if they have a serious condition or terminal illness. The challenge referred to in these articles is how to address a patient’s stated wishes not to be resuscitated if the patient needs palliative surgery; see Table 3.

**Table 3:** Case illustration: DNR orders in the OR

*A Professor A. Droit, 93 years of age, formerly your college ethics teacher, developed a painful ischemic foot from distal aortic blockage. A daughter, who is a nurse, brought him to the hospital. He has multiple comorbidities, including leukemia for which he is getting chemotherapy. He agrees to surgery but hands you a completed do not resuscitate (DNR) form and insists it be honored throughout his care. As the operative wound is being closed, he has a slow ventricular tachycardia, which does not respond to intravenous therapy.*	
Jones [39]	

Highlighted in many of the studies, a central argument for suspending the DNR order while the patient is in the OR, is that the survival rates for resuscitation are significantly greater [19–29]. The authors describe how a cardiac arrest in the OR will be immediately recognised and trained personnel as well as defibrillators and medication are directly at hand. Furthermore, many authors point to the fact that cardiac arrest in the OR is often a direct result of the intervention, as it is an intrinsic part of anaesthesia or surgery to meddle with vital organs and body functions. Continuous artificial ventilation is, for instance, a prerequisite for general anaesthesia. The lines between anaesthesia and resuscitation are expressed by many authors as rather artificial and obscure [20–23, 27, 30–34].

As a consequence of the highly specialised environment in the OR, several authors suggest that patients’ DNR orders may not be valid for the OR setting [21, 23, 26, 28, 32, 33, 35]. Nevertheless, others hold that the issue is primarily patient autonomy and that it is paternalistic to override patients’ decisions [21, 22, 24, 36–42]. Several authors draw a parallel to Jehovah’s Witness, addressing the issue of restricting anaesthesiologists’ and surgeons’ range of action [25, 42–45]. Similar issues are also reflected in the discussion of guidelines, which have developed from the practice of automatically suspending the DNR order in the OR to “required considerations” in the preoperative patient consultation [20, 22, 24, 36, 42, 44, 46–48].

Some of the publications focus on the surgeons’ special responsibility for the outcome and the well-being of the patient, as surgeons are often seen as in command of the OR. Accepting a DNR order in the OR is repeatedly portrayed as opposing surgeons’ professional roles and duties [22, 25, 33, 34, 44, 45, 49–51]. Several authors describe the issue of responsibility as pertinent since cardiac arrest may be caused by anaesthetic or surgical intervention. They contend that surgeons may feel particularly liable if the patient is allowed to die of an iatrogenic complication in the OR [21, 22, 25, 26, 42, 46, 52].

More existential challenges are also expressed in lines of the OR being a poor location for deaths [20–22, 25, 32, 33, 44, 45, 52]. This is stated in terms of disadvantages for patients, being removed from their families and loved ones, and for OR personnel who are exposed to stress and negative emotions. Death in the OR is considered a “bad outcome”, which may cast suspicion on the personnel and initiate formal investigations.

### Overlapping surgery

A frequent topic in the reviewed publications is overlapping surgery. This phenomenon refers to the practice of allowing a lead surgeon to be in charge of two ORs at the same time. In these cases, while junior surgeons or surgeon trainees are present for the entire operation, the head surgeon performs or oversees only the most important or difficult parts of the surgical procedure.

Efficiency is a central argument for allowing overlapping surgery according to many of the reviewed studies. When each leading surgeon handles two ORs, the OR teams and resources can be used more efficiently, and more surgeries can be performed [53–55]. OR efficiency is mentioned in relation to surgeons’ career advancement, as surgeons need high-volume surgery [53, 56, 57]. It is also argued that OR efficiency benefits patients, as they get improved access to health care and high-impact surgeons [53, 55, 58]. Another argument in favour of overlapping surgery that appears in several of the publications is that it allows junior surgeons to perform more advanced operations under expert supervision. Thus, overlapping surgery is required to train surgical residents [53–56, 59].

Many of the reviewed articles discuss the potential risks of overlapping surgery. The risks are described in terms of more inexperienced surgeons, increased complication rates and surgeons’ loss of focus [55, 58, 59]; see Table 4. Some authors question whether this practice entails augmented risks and advocate further consideration [57, 60]. In the publications, discussion of risks is often linked to the definition of overlapping surgery, where the surgeon must be present in the OR for the “critical part” of the procedure [58, 60, 61].

**Table 4:** Case illustration: overlapping surgery

*On August 7, 2012, Tony Meng, a financial analyst and father of two from Westwood, Massachusetts, underwent a cervical corpectomy performed by Dr. Kirkham Wood, an experienced and respected orthopaedic spine surgeon at MGH. Mr. Meng was Dr. Wood's second case of the day. Meng was put to sleep at 8 am just after Woods started his first case. During Meng's case, Wood made six trips in and out of the operating room as he also attended to the first patient. While performing Meng's corpectomy, the monitoring signals faded and at 1:30 pm, all muscle responses were lost. Following the corpectomy, Wood went back to his first case and then returned to Meng's case to perform cervical laminectomies on him. Meng's case was finished at 7:30 pm, at which time Wood went on to his third case of the day. An MRI done on Meng late in the evening showed the cervical spinal cord at an acute angle, and he was returned to surgery after midnight where a large anterior dural defect was found and repaired. Meng is now quadraparetic and wheelchair bound.*	
Scarrow [55]	

In addition to the risks of complications, the issues of responsibility and trust are mentioned by several authors. Some studies describe how overlapping surgery may violate head surgeons’ responsibility to patients because they are liable for the whole operation [53, 58, 61]. Even more studies thematise the special relationship of power imbalance and trust between patient and surgeon, which should be respected, often with referral to patient consent [53, 57–61].

### Donation of organs

Organ donation, including donation after brain death (DBD) and donation after circulatory death (DCD), is a recurrent topic in our review. While brain death is the traditional ground for initiating organ donation, many states have more recently opened for DCD. DCD requires death to occur in a relatively controlled manner, often inside the OR, as organs must be retrieved immediately after the time of death. The included publications address the topics from within the OR, that is, the surgery and the decisions involved after patients are cleared for organ retrieval procedures.

All of the publications on organ donation deal with the ethical challenge of balancing the potential benefit for the recipient patient with the potential harm for the donating patient [62–68]. Several studies describe the possibility of harming donor patients at the end of life [64–67]. At the same time, the need to minimise delay after death to secure viable organs is accentuated [62, 64, 65].

Indeed, the very shift of focus from care for dying patients to care for their organs is expressed as challenging by several authors [63–68]. It is considered ethically conflicting that the donor’s welfare is not the rationale behind the surgery. Several studies describe what is interpreted as a lack of respect and dignity for the donor patient [65–68].

Adding to the ethical conundrum, some publications reveal a sense of uncertainty around donor patients’ deaths because our notions and definitions of death are challenged by the fact that the patients’ organs are defined as viable [62, 64–67].

Organ donation is described as a challenging experience for OR personnel in most publications on the subject [62, 63, 65–68]. This is both explained by the fact that the occurrence is uncommon and outside their field of expertise [62, 63, 65, 67, 68] and because their actions appear contrary to their professional duties [63, 65–68] (see Table 5).

**Table 5:** Case illustration: donation of organs

*A COMMUNITY hospital agrees to participate in organ harvest from non–heart-beating cadaveric donors (NHBCDs). Members of the anesthesiology department are informed that patients requiring life support will be transferred to the operating room, where an anesthesiologist will monitor them during preparation and draping for organ harvest. The anesthesiologist will discontinue life support and administer medications to keep the patient comfortable while he or she dies. Three minutes after asystole ensues, the anesthesiologist will pronounce the patient dead, and organ harvest will immediately begin.*	
*The anesthesiologists question the ethics of stopping life support and then harvesting vital organs. Some believe it is acceptable to discontinue life support and administer medications to stop respirations and hasten death. Many are resentful that an unpleasant task is being thrust onto them by other physicians in a manner reminiscent of “orders to nurses.” Most express bewilderment that the duties of discontinuing life support, caring for the dying patient, and diagnosing and pronouncing death should fall to an anesthesiologist.*	
VanNorman [67]	

### Broadcasting live surgery

Live surgery demonstrations are a traditional way of learning new operation techniques. With new technological advances, live surgery can now be broadcast to a large audience across the world. Live surgery broadcasts have been highly popular additions to surgical conferences in a variety of disciplines, but medical societies have gradually become more sceptical of this practice, as is evident in our review.

A central rationale behind live surgery broadcasts, as addressed by some of the studies, is effective knowledge dissemination and advantages to fellow colleagues [69–71]. However, some questions remain as to whether live surgery has any advantage over recorded video surgery [72–74]. Another rationale for live surgery, apparent in our review, is patient benefits. Patients may obtain access to expert surgeons, advanced equipment, and therapies they would otherwise not afford [69–71].

Nevertheless, concerns about patient safety are prominent in our reviewed studies. Several components of patient safety, such as increased risk of infection [69–71], surgeon distraction [69–72, 75] and operating in unfamiliar environments [70, 71, 75], are discussed. Some also address potential violations of confidentiality and patient dignity [71, 75]. Many studies address the psychological stress of surgeons performing live operations [69–71, 73, 75]. The OR and equipment may be unfamiliar, there may be language barriers, and surgeons may be exposed to jetlag, time pressure and the expectations of the crowd.

Many of the authors describe a challenge of conflicting interests [69–72, 74, 75]. According to them, broadcasting live surgery serves multiple purposes in addition to healing the patient, such as educating and entertaining the audience, promoting an expert surgeon, promoting a private institution, or advertising medical equipment (see Table 6).

**Table 6:** Case illustration: broadcasting live surgery

*A few years back, I participated as an organizer in a workshop on liver surgery. A leading surgeon from abroad was invited to demonstrate a resection of the liver. A huge tumour of the liver, the removal of which would in any case be controversial in terms of benefit to the patient, was given as a ‘challenge’ to the surgeon. The surgeon, who had arrived late at night, was escorted straight into the operation theatre the next morning. He was also a little ‘taken aback’ by the size of the tumour but chose to go ahead with the procedure. The surgeon demonstrated what he claimed was a ‘bloodless’ and ‘quick’ method of removing the tumour by clamping all the blood vessels to the liver and cutting through the liver with a knife. A spellbound audience cheered at the end of the procedure and the surgeon triumphantly came to the stage and waxed eloquent about his method. A few hours later, the patient bled massively and had to be re-operated in the evening by the local team, the surgeon having moved on to other commitments. Unfortunately, the patient succumbed some days later. By that time, the workshop was over and the audience was completely unaware about the complication and sequence of events, which are, in fact, a known potential hazard of the method used by the surgeon.*	
Nagral [74]	

Interestingly, many of the publications discuss the practice of live surgery broadcast in relation to the very nature of surgery. Some claim that broadcasting converts surgery into dramatic performance and thrilling shows [72, 74] and that it hampers the surgeon’s core responsibility for the patient and for actions within the OR [70, 74, 75]. Others hold that broadcasting the performance accentuates what surgery is about—the power and vulnerability of the surgeon, immediate decision-making, creativity, and apprenticeship [70, 73].

### Video recording

With technological advancements and as recording devices have become increasingly smaller and less expensive, there has been a growing inclination to include recording devices in the OR, a topic that is repeatedly mentioned in the reviewed articles. Video recording may serve different ends, as evident in the review.

A common argument to allow recording in the OR is that it leads to quality improvement, as surgeons may review and learn from their mistakes [76–80]. Recording is also described as an effective mode of teaching, making the operation field more accessible to colleagues, junior doctors and even patients [76, 78, 79, 81]. Furthermore, being recorded and potentially observed may have a positive effect on the behaviour of the surgeons and the OR team, according to some authors [77–79]; see Table 7. However, a few note that the knowledge of being watched may also add stress to the surgeon and thus negatively affect performance [78, 79].

**Table 7:** Case illustration: video recording

*A third of subjects believed that derogatory and unsavory comments are commonly said about patients in the operating room. Subjects referenced news reports in which healthcare providers were accused of and/or caught behaving unprofessionally: “There were some pretty crude things that were said about a patient” (V34); “Well, I mean, there was that example of the surgeon who was dancing and filming her patients, and things like that” (V05). Some subjects believed that being recorded would “keep the doctors on their toes” (V2) and discourage them from engaging in unprofessional behavior or commentary—a net benefit*.	
Gallant [78]	

Several studies describe how video recording may add a desired transparency to an area of medicine to which few people have access and that recordings may play a role in litigations [77–80]. The ownership of the recorded data is also discussed, related to data editing or erasing, as well as distributing or selling data [77, 78, 80, 81].

A central argument against video recordings in the OR, as evident in our review, is possible breaches in confidentiality or privacy. The possibility of inadvertently sharing patient identifiers is discussed in all publications on the subject [76–81]. This may be anything from a written or spoken name, parts of a face, a unique tattoo, or even discussions of other patients during the operation. Some studies also address the potential violation of privacy of OR team members, who may share personal information while working together and who are not easily anonymised [77, 79, 81]. The need for informed consent from patients, and possibly OR team members, was mentioned by several authors [77–81], although one paper explicitly stated that the surgeon should not be allowed to decline because of the recording’s possible benefits to patients [79].

### Communication and teamwork

Several of the articles in our review discuss how OR personnel work together as a team. The ethical value of a well-functioning OR team and the communication between team members are explored in these articles. Several studies refer to a traditional view with the surgeon as “captain of the ship”, dictating other team members at will [82–84]. This is described as an outdated and egocentric concept, indicating character flaws. Instead, valuing and respecting each other’s roles in the team is accentuated by some authors [82, 85, 86]. A few among these especially mention how this is central to avoiding conflicts between surgeons and anaesthesiologists [85, 86]; see Table 8. Attention to the team’s common goal of a favourable outcome is seen as a way to overcome professional differences [83, 84, 86].

**Table 8:** Case illustration: communication and teamwork

*On a busy elective [operation theatre] day, onco-surgeon was going to operate a 70-year-old patient of carcinoma thyroid with neck metastasis. Other routine cases were also on the list. Anesthesiologist requested the chief of the surgery to allow the onco-surgeon to operate on a table having invasive monitoring as the case is complicated. Surgeon scolded at the anesthesiologist “who are you to decide that which case is to be operated on which table” and he was not willing to listen to anything and was using degrading language. In view of patient’s safety intervention from the chief of anesthesiology was sought, and patient was put on invasive monitoring table. Now, in this case, surgeon behaved in a manner as if he was the “captain of the ship” and was a cause of conflict. But firm stand taken by the chief of anesthesia in favour of patient safety was again an example of collaboration.*	
Attri [85]	

The need for a positive work environment in the OR is accentuated in all the articles on this theme [82–87]. A positive work environment is advocated to reduce work stress and improve patient safety. Interdisciplinary collaboration, effective communication, collegial relationships and a flat hierarchy are described as ways to achieve this.

Some authors also address the practical nature of the work inside the OR, attending to ethical virtues [82–84]. According to these authors, surgeons should be aware of their position as role models, team members should strive for high professional levels, and beneficial team practices should be routinised.

### Implementing new surgical technology

A few of the studies in our review deal with issues related to new surgical technology or techniques. While they cover a variety of technologies, the studies address some common moral challenges. The technology in question ranges from new aseptic techniques in 1976, via robotic surgery, to mixed reality glasses in 2022.

A question that is addressed by many of the authors on this subject is who bears responsibility when new technology is introduced in the OR [88–91]. The surgeon’s personal responsibility is stressed while also acknowledging the significance of team effort and the difficulties associated with assigning responsibility when operating non-transparent machinery. Some authors also relate the question of responsibility to legal regulations and lack thereof [89, 91].

Several articles address how new technology may change surgeons’ self-conception and autonomy [89–92]. The shared responsibility of a robot, the technological assessment of surgical performance, the need for new skillsets and the loss of traditional surgical skills are all issues discussed here. Another challenge mentioned in the articles is the uncertainty and ever-evolving understanding of new technologies [89–91]; see Table 9. Rules need to be constantly revised, empirical and theoretical questions are evolving, and many human-technological interactions lack objective criteria.

**Table 9:** Case illustration: implementing new surgical technology

*In 1956 anyone predicting a person would be walking on the moon would have had their sanity questioned, yet little more than a 231 decade later it was an established fact. In 1989, no one would have considered using a robot to perform surgery on a patient-laparoscopic surgery was emerging as the revolutionary change. However, in 8 years (April, 1997) the first robotic cholecystectomy was performed in Brussels by Himpens et al.2 The long-term as well as the fundamental implications of this event have yet to be seriously considered, let alone the moral and ethical implications.*	
Satava [90]	

All reviewed articles on this subject refer to possible risks or benefits to patients [88–92]. The dissemination of sensitive data, unknown risk in new procedures and incorrect programming are among the potential harms described. High-quality surgery, better performing surgeons, and new possibilities to explain and include patients and caregivers are among the potential benefits described. A possible conflict of interest between patients and surgeons in allowing new techniques in the OR is also expressed [89, 90].

### Denying blood to Jehovah’s Witness

In many Western countries, Jehovah’s Witness have a legal right to refuse blood transfusion. Withholding blood transfusion from a bleeding patient in the OR might lead to serious medical conditions or even death. This moral challenge is addressed in several of the reviewed papers.

All of the papers on the issue describe this as a conflict between beneficence (doing good) and respecting patient autonomy [93–96]. Beneficence would here imply improving patients’ medical conditions and providing blood transfusions to patients according to their needs. Respecting patient autonomy would mean honouring a patient’s request to abstain from blood transfusion, even if it might jeopardise the operation and patient recovery.

According to the included papers, American patients have a legal right to forgo blood transfusion. Nevertheless, this will not always settle moral difficulties. One of the challenges mentioned by several authors is that the patient’s wishes are not always unambiguously stated. This means that the surgeon must morally assess how clearly the patient’s preferences are known or how possibly to substitute the patient’s consent [94–96]; see Table 10. Some of the studies also discuss how to interpret beneficence, or the best medical practice, when blood transfusion is not an option. The use of other fluids, alternative preoperative management and intraoperative precautions might reduce the risk of threatening blood loss [94, 96].

**Table 10:** Case illustration: denying blood to Jehovah's Witness

*An elderly woman was brought to the emergency room (ER) hypotensive in a confused mental state from what turned out at exploration to be a ruptured splenic artery aneurysm. You are in the operating room, and the anesthesiologist has just hung the first unit of blood but has not started infusion when the ER calls. The patient and her husband were visiting their children and live in another state. Her husband, an elder in a Jehovah’s Witness congregation, arrived and is adamant that she have no transfusions. Her blood pressure is dangerously low. It is being maintained by a high-dose Levophed (leave-um dead) drip and continues to slip. You have avoided operating on Jehovah’s Witness patients because of the added unnecessary risk they pose. Your assistant is of like mind. What is the best ethical course at this time?*	
Jones [95]	

### Other OR challenges

Almost one-third of the publications in our review deal with issues other than those mentioned above. These studies address a variety of topics that are not easily categorised, varying from surgical checklists to moral challenges of obstetric surgery in the 19th century.

Some authors present an overview of moral challenges in surgical ethics, covering different topics in a single paper [97–99]. A few address how some moral phenomena are distinctive for surgical speciality, such as the responsibility for actions and the nature of the patient relationship [100, 101]. Many of the studies cover issues related to professionalism and surgeons’ moral character [102–109]. These are descriptions of adequate virtues and behaviour for surgeons, as well as examples of unprofessional conduct. Possible conflicts of interest are thematised in some studies, describing how the surgeon or other personnel inside the OR might have other aims than benefiting the patient [110–114]. Additionally, several authors address the conflict of interest entailed in surgical training, using the patient partially as a means to improve junior doctors’ skills [115, 116]. The close link between patient safety and ethics is revealed in a few articles addressing medical errors induced by individual surgeons and by institutions [117–119]. Interestingly, quite a few authors speak of a challenging tension between the humanness of patients or OR teams and the dehumanising conditions of the OR [120–126]; see Table 11. This tension could manifest itself as undignified patient care or as a trauma to OR team members experiencing the death of a patient.

**Table 11:** Case illustration: other OR challenges

*James O'Hara (the last name is fictional) was three days old. On his first day of life, he had developed cyanosis and heart failure, and was diagnosed as having hypoplastic left heart syndrome. (…)*	
*My role as surgical liaison nurse includes letting the family know how the surgery is going and to provide emotional support. The surgeon asked the OR nurse to have me tell the parents the baby was having a problem, and I did so. Soon, I had to deliver more bad news- that nothing was working, that despite the OR team's best efforts, James probably wouldn't survive the operation. The child's parents and maternal grandmother reacted as might be expected- crying, praying, and holding each other. Clearly, they understood the gravity of the situation. His mother pleaded to be allowed into the OR to say goodbye to James while he was still alive. I explained that he was under deep anesthesia, that his chest was open, that there were IV lines everywhere. She said she understood but felt compelled to be with her baby. (…)*	
*I knew that when a patient does die in the OR, the staff is under tremendous stress. Each team member feels a sense of responsibility. To let Ms. O'Hara enter the OR, with no preparation or planning, would have been unbearable for the staff, I thought.*	
Fina [121]	

## Discussion

We performed a systematic review of scientific publications on the ethics of surgery, which is a rather new and evolving branch of medical ethics. Very few systematic reviews have been conducted on surgical ethics, and most have focused on surgical training and surgical innovation [127–130]. To our knowledge, there exists no systematic review on ethics in the operating room. We found that less than 2% of the publications that we identified on surgical ethics addressed activities or events inside the operating room (OR). This result raises concerns, as the OR is considered to be the main workplace for surgeons. Additionally, one would expect the act of surgery to be a prominent feature of the ethics of surgery since this is the feature that mostly differentiates it from other medical specialities. There may be several reasons for this relative neglect. One reason may be practical: the OR is a confined area that is not readily accessible to people outside the operating team. This makes it harder for researchers to gain access, and it also makes it more remote for medical ethicists. Thus, the reason for the lack of ethics papers from within the OR may also be epistemological; what is out of sight is also out of mind. Many ethicists may not be aware of what takes place inside the OR and thus have not reflected on the ethical aspects of this activity. Surgeons normally address their work inside the OR in technical terms and are often not accustomed to think of the ethical aspects of surgery. It is possible that the present body of scientific publications on the ethics of surgery may, in effect, conceal ethical issues inside the OR.

At the same time, this systematic review indicates that there are important ethical issues inside the OR. The most salient issues in the included publications were DNR orders in the OR, overlapping surgery, donation of organs, broadcasting live surgery, video recording, communication and teamwork, implementing new surgical technology and denying blood to Jehovah’s Witness. These eight topics were the main focus of two-thirds of the included papers, and the issue of DNR orders in the OR was addressed by one-third of all included papers. A reason for such a pooling of topics may be that once an ethical challenge is recognised, it is easier for others to address. Additionally, since we did not restrict our search to scientific articles and included editorials and comments, an issue discussed in a scientific journal might have led to several responses and thus increased the number of corresponding papers in the review. Only 10% of the included publications were original research articles.

The included articles display what was identified as ethical questions inside the OR. Not surprisingly, the review shows that different ethical principles are at stake in the described situations, and the four principles of biomedical ethics were frequently cited [4]. The American dominance of the literature may also have affected the issues that were addressed. More than two-thirds of all included papers were from a US context, which may reflect that the ethics of surgery is a more developed discourse in this area and indeed has its origins here [5]. Additionally, patient autonomy has a strong hold in American law and health care practice, which was reflected in the discussions, especially on topics such as DNR orders in the OR and denying blood to Jehovah’s Witness [44, 96]. Interestingly, our findings show that the issue of autonomy is often more composite inside the OR because health care personnel might question whether the patient’s decision is sufficiently informed to be autonomous. The OR is a complex environment, and surgical procedures may be intricate in such a way that patients may not fully understand what they consent to, a challenge clearly illustrated by the discussion on DNR orders in the OR [33].

Medicine and health care are areas of rapid technological development. New scientific inventions combined with caring for patients often spark ethical debate [131]. This was reflected in our review, where ethical concerns about technological development were linked to a diversity of patient safety issues. Unknown risks in new procedures were addressed by several authors, as equipment or methods not yet have undergone rigorous clinical testing: “Technology is changing faster than the profession can react”, citing an author discussing laparoscopic surgery [90]. Unfamiliarity with the equipment was another frequent topic discussed in relation to patient safety [70]. Additionally, the issue of increased risk due to surgeon stress was addressed in some of the papers, for instance, when surgeons were preoccupied with mastering new technologies in broadcast surgery [69]. An ethical issue often discussed in relation to patient care and scientific advances was patient confidentiality. Several papers addressed the challenge that new technologies often include electronic registration of data that has the potential for spread outside of the confined OR area. This concern was especially raised in articles on audio and video recording technology, and one study showed that patient identifying information was exposed 1.13 times per minute during the operation [76]. The risk of patient confidentiality violations was also addressed in relation to the possible dissemination of other logged patient data, especially the use of Big Data combined with sensitive information [91].

Another issue commonly raised in the included articles was that of conflicting interests. This was emphasised in the discussion on organ donations, where it sometimes proved difficult to manage the interests of the donor and the recipient equally [63]. Patients and doctors may also have diverging interests that may affect care, as shown in the case of overlapping operations where junior surgeons need to obtain the necessary skills [54]. Furthermore, the review revealed that surgeons and anaesthetists may have different work codes and interests in surgical cases, not always agreeing on who is in charge of what [86]. Other interests may also be represented in the OR, affecting the surgery, as illustrated in the review. Salespersons who interact or even help surgeons in the OR visualise tight company interests in certain specialties [111]. Video recordings are often used for the benefit of educating junior doctors or colleagues [76] but might also be used as a means for hospital administration to monitor OR staff behaviour [79].

A more latent ethical feature that emerged through many of the articles was the tension between the sterile and objectivating conditions of the OR and the humanness of the patients and the people working there. While rarely addressed explicitly, the notion was expressed through statements such as “OR is no place to die” [52] or “we must restore the humanness of the patient” [120]. The fact that members of the OR team were also affected by this tension was explained in several articles. It was characterised as “unbearable” for the staff to let the mother of a dying baby inside the OR [121] and turning off the ventilator of an organ donor patient was described as “a traumatic experience” [63]. While these examples show that the staff were experiencing tension between OR objectivation and humanness, other studies have shown that neglecting this tension could have unethical consequences. Some authors described how staff contributed to patient humiliation by adhering to their medical tasks and ignoring patients’ voiced or nonverbal expressions [126].

The notion of surgeon responsibility was frequently addressed in the included articles. Several papers stressed that, opposed to times passed, the surgeon is no longer “captain of the ship” [82–84]. Nevertheless, the special responsibility of the surgeon was underlined by many authors, and the reviewed papers displayed how key decisions are made by surgeons. This included medical questions, such as defining the critical portions of a procedure [60], and more conceptual queries, for example, deciding whether a patient request not to be resuscitated is, in fact, applicable [33]. The responsibility of the surgeon was, in several articles, depicted as a personal obligation. Some authors have linked this to the nature of surgical actions because these are invasive and entail risks of serious harm [2, 91]. Others have discussed personal responsibility in relation to the special bond between surgeons and patients, for instance, referring to surgeons’ “required considerations” in preoperative patient consultations on DNR orders [22]. According to the description of legal regulations in some countries, the surgeon has a contractual responsibility even for mistakes made by other members of the OR team [101].

### Strengths and limitations

A strength of this systematic review was the large number of articles included in our analysis. This gave us an opportunity to show the diversity in the field and provide a scope of what ethical issues are addressed from inside the OR. We did not restrict the search to any specific time period, adding to the diversity. We included both scientific articles, editorials and brief comments, as we were interested in all accounts of ethics from the OR. This added to the diversity, but on the other hand, it opened up to less theoretically or scientifically founded papers. Very few systematic reviews have been conducted in the field of surgical ethics, and we have complied with the 22 requirements for reporting of systematic reviews in ethics (RESERVE), which build on the PRISMA and ENTREQ guidelines [132], see Additional file 4 for the complete checklist.

The literature search was only conducted in Medline and Embase, and a search in multiple databases and grey literature might have provided different perspectives, which were not included in our review. However, Medline and Embase are the largest and most inclusive databases for medical issues, and we assessed that they would cover most of the publications in question. We restricted the search to English and Scandinavian languages and thus excluded studies published in other languages.

The definition of concepts necessarily affects the outcome and defining “ethics” is not straightforward. This is especially true in the field of medicine, where you could argue that all acts of helping patients are within the field of ethics. Additionally, a large number of articles addressed issues very close to ethics, such as quality improvement, communication or law. Because we were interested in the ethical issues in the OR that had been raised and explicitly discussed, we decided to include only articles that openly discussed ethical questions. In doing so, we might have missed potentially interesting articles, perhaps especially from doctors and surgeons, who rarely discuss medical issues in ethical terms.

Defining what is “inside the OR” turned out to be slightly more challenging than originally thought. While the confinement of the OR itself is quite sturdy, many decisions take place partly outside, before the operation, and partly inside, during the operation. The question of whether to respect a DNR order in the OR is closely linked to the preoperative discussion with the patient. Surgical checklists are made and implemented in the organisation outside the OR but are enacted on the inside. Mostly, the distinction of outside/inside the OR was evident. For the debatable cases, we included the articles if *some* of the ethical actions described took place inside the OR, thus erring on the side of inclusion.

It is worth noting that surgeons were the focus of our enquiry, which probably affected our outcomes. While surgeons are mandatory in surgical procedures, many activities inside the OR are conducted by other health care personnel, such as anaesthetists, nurse anaesthetists, surgical nurses and circulating nurses. Focusing on other health care personnel might have accentuated other ethical challenges inside the OR.

## Conclusions

The ethics of surgery is a relatively new field within medical ethics that needs further exploration. Our systematic review revealed that a relatively small number of studies on surgical ethics have addressed ethical issues inside the operating room (OR). The ethical issues that were presented from the OR were manyfold, although most of the included articles addressed the same issues. This might indicate that once a situation is identified as moral, it is more readily debated in public. If so, this calls for exploratory work on the ethics of surgery, acknowledging the special realities and circumstances of surgeries and ORs, and acquiring the sensitivity required to raise the moral issues that are embedded in this medical work. In the discussion, we have pointed to some possible issues to probe: the meaning of patient autonomy inside the OR, the consequences of technological advances in surgery, the balancing of legitimate interests, the dehumanising conditions of the OR, and the strong notion of surgeon responsibility. Identifying the full scope and peculiarities of an ethics of surgery will benefit patients receiving surgery and may also support OR professionals, especially surgeons, standing in the midst of hard moral choices at work.

## Supplementary Information

Supplementary Material 1.

Supplementary Material 2.

Supplementary Material 3.

Supplementary Material 4.

## Data Availability

All data generated or analysed during this study are included in this published article and its supplementary information files.

## Abbreviations

OR: Operating Room
DNR: Do-Not-Resuscitate
DBD: Donation after Brain Death
DCD: Donation after Circulatory Death
