# Supplementary material for: Ethics in the operating room: a systematic review
Source: BMC Med Ethics. 2024 Nov 9;25:128. doi: 10.1186/s12910-024-01128-7 (PMC11550563; doi:10.1186/s12910-024-01128-7)
Supplement: Supplementary file 1 — Supplementary Material 1. [file 12910_2024_1128_MOESM1_ESM.docx]

| **Search string Medline** | | |
| --- | --- | --- |
| **#** | **Query** | **27.04.2023** |
| 1 | exp Specialties, Surgical/ or exp Surgeons/ or exp Surgical Procedures, Operative/ or exp Postoperative Complications/ or exp Surgical Equipment/ or exp Orthopedic Procedures/ | 3,954,058 |
| 2 | (surger* or surgic* or surgeon* or surg* complication* or operatively* or operative* treatment* or operative* management* or operative* technique* or operative* procedure* or operative* time* or operative* field* or preoperative* or pre-operative* or postoperative* or post-operative* or perioperative* or peri-operative* or intraoperative* or intra-operative* or reoperation* or re-operation*).ab,ti,kw. | 2,657,944 |
| 3 | (operation* or operative*).kw. | 6,072 |
| 4 | 1 or 2 or 3 | 5,094,984 |
| 5 | exp Morals/ or exp Ethics/ | 182,797 |
| 6 | (moral* or ethic*).ab,ti,kw. | 190,447 |
| 7 | ethics.fs. | 76,887 |
| 8 | 5 or 6 or 7 | 326,731 |
| 9 | exp Operating Rooms/ | 15,764 |
| 10 | (operat* room* or surg* room* or operat* theat* or surg* theat* or surg* facilit*).ab,ti,kw. | 43,610 |
| 11 | 9 or 10 | 50,259 |
| 12 | 4 and 8 and 11 | 539 |
| 13 | limit 12 to (danish or english or norwegian or swedish) | 513 |

| **Search string Embase** | | |
| --- | --- | --- |
| **#** | **Query** | **01.05.2023** |
| 1 | exp Surgery/ or exp Surgeon/ or exp Postoperative Complication/ or exp Surgical Equipment/ | 6,523,647 |
| 2 | (surger* or surgic* or surgeon* or surg* complication* or operatively* or operative* treatment* or operative* management* or operative* technique* or operative* procedure* or operative* time* or operative* field* or preoperative* or pre-operative* or postoperative* or post-operative* or perioperative* or peri-operative* or intraoperative* or intra-operative* or reoperation* or re-operation*).ab,ti,kw. | 3,842,584 |
| 3 | (operation* or operative*).kw. | 12,514 |
| 4 | 1 or 2 or 3 | 7,408,286 |
| 5 | exp Morality/ or exp Ethics/ | 383,088 |
| 6 | (moral* or ethic*).ab,ti,kw. | 276,824 |
| 7 | 5 or 6 | 511,751 |
| 8 | exp Operating Room/ | 53,093 |
| 9 | (operat* room* or surg* room* or operat* theat* or surg* theat* or surg* facilit*).ab,ti,kw. | 65,399 |
| 10 | 8 or 9 | 82,843 |
| 11 | 4 and 7 and 10 | 1,768 |
| 12 | limit 11 to conference abstracts | 633 |
| 13 | 11 not 12 | 1,135 |
| 14 | limit 13 to (danish or english or norwegian or swedish) | 1,072 |
