# Supplementary material for: Ethics in the operating room: a systematic review
Source: BMC Med Ethics. 2024 Nov 9;25:128. doi: 10.1186/s12910-024-01128-7 (PMC11550563; doi:10.1186/s12910-024-01128-7)
Supplement: Supplementary file 2 — Supplementary Material 2. [file 12910_2024_1128_MOESM2_ESM.docx]

# **Research questions:**

1. How much of the scientific publications on surgical ethics focus on what take place inside the operating room?
2. Which ethical issues are covered by the publications that focus on ethics in the operating room?

## **PICO:**

### Populations = Surgeons

#### Intervention = Actions and decisions taken inside the operation room (OR)

#### Comparison = Any or none

#### Outcome = Ethical aspects emerging from actions and decisions inside the OR

**Evaluation of significance:**

Articles with main focus on ethical issues that unfold inside the OR and addresses surgeons. The ethics should be explicitly stated and treated in some depth.

|  | **Author/**  **year/**  **country** | **Title** | **Type of article** | **Issue/Research question** | **Main findings relevant for our review (ethics of surgery inside the OR)** | **Significance** |
| --- | --- | --- | --- | --- | --- | --- |
| **1** | Angelos,  2016,  USA | The Ethics of Introducing New Surgical Technology Into Clinical Practice. The Importance of the Patient-Surgeon Relationship | Comment  2p | Patient information and consent related to implementing new surgical techniques | - The patient bears the risk involved in new procedures (not the surgeon)  - Insists on the surgeon’s personal responsibility:  - The surgeon bears ethical responsibility for solving unexpected events in the  OR for the benefit of the patient | Minor  - Consent |
| **2** | Angelos,  2019,  USA | “Clinical” Surgical Ethics | Review article  7p | Show how the phenomenology of surgical ethics differs from general medical ethics | Phenomena that are distinctive to surgical ethics:  - Responsibility: close relation between actions and outcome  - Surgeon-patient relationship: Requirement of harm, trust – encompassing the  whole surgical team, physical intimacy, vulnerability  - Expectations to use innovative techniques for the patients’ benefit  - Unexpected findings and the need to decide on behalf of the patient  - Training of new surgeons while maintaining patient safety | Substantial |
| **3** | Angelos,  2020,  USA | Responsibilities and Expectations. Considerations of Disclosure of Overlapping Operations | Comment  2p | 2 central ethical questions in overlapping operations: 1) Are they inherently unethical?  2) Should surgeons always disclose this to patients? | - Division of labour is characteristic of advanced procedures  - Overlapping procedures increases the surgeon’s expertise  - The surgeon has a responsibility to be as experienced as possible  - The anaesthesiologist relationship with the patient is more remote | Minor  - Consent |
| **4** | Attri,  2015,  India | Conflicts in operating room: Focus on causes and resolution | Review article  7p | Quality of patient care depends on effective teamwork and communication between anaesthesiologists and surgeons | - Possible conflict of interest between anaesthesiologist and surgeon  - Possible ethical conflicts in the OR: informed consent, treatment plans, degree of  preoperative tests, directives that limit treatment or DNR orders  - No hospital policies to regulate anaesthesiologist-surgeon relationship  - Fear of litigation and malpractice claims  - Occurrence of preventable medical errors  - Decreasing work performance | Minor  - Quality of care |
| **5** | Bagwell,  1992,  Canada | Spinning the Wheels: A CAPS Survey of Ethical Issues in Pediatric Surgery | Quantitative article  6p | Questionnaire with 16 questions (15 ethical dilemmas in a clinical setting), to evaluate decision-making factors of paediatric surgeons | - HIV patient: Duty to care. Risk of exposure.  - Parents to Jehovah’s Witness child cannot deny urgent need for transfusion.  - DNR order affects treatment plan  - Operate on newborn with fatal condition even if no real prospect of prolonged life  (no operation mortality) | Minor  - Outside OR |
| **6** | Bakhtiari,  2020,  Iran | Perspective and Experience of Operating Room Personnel on Ethical Behaviors | Qualitative article  10 p | Interviews with OR personnel to assess experience of ethical behaviour.  (Only 3 doctors of 26 participants) | - “Ethical behaviour” as opposed to moral reasoning, decision-making and dependent on context-specific setting. Assess experience of OR personnel.  - “What is your understanding of ethical behaviour in the OR”  - Main themes:  - Professional commitment; clinical skills, accountability, job discipline  - Strive for excellence; character, teamwork, knowledge of patients/relatives  - Respect for human dignity; individuality, fairness to patients and to staff  - Commited to enhance safety; calm atmosphere, avoid abuse, protect/prevent | Minor  - Nursing |
| **7** | Barnes,  2012,  USA | Practicing Pelvic Examinations by Medical Students on Women Under Anesthesia.  Why Not Ask First? | Personal narrative  3p | The practice of unconsented practice pelvic examinations by medical students on women under anesthesia is common and unethical | - Pro-arguments refuted (inside OR):  - Minor activity as part of surgery vs intrinsically different task  - If medically warranted – then students are prohibited from participation | Minor  - Consent |
| **8** | Bartlett,  2015,  USA | Do-not-resuscitate. Providing ethical care and promoting patient independence | Review article  4p | Nurses should be involved early in the decision-making process to advocate for patients in making appropriate decisions regarding DNR status. | - The nurse as the patient’s advocate  - Suspension of DNR in OR: ill-defined area. Lack clear hospital policies  - General anaesthesia requires intubation, ventilation  - The burden rest on the surgeon and the anaesthesiologist | Minor  - Nursing |
| **9** | Berger,  2015,  USA | Creating a Humanistic Surgical Theater: The Patient-Centered Pause | Comment  < 1p | Introducing a statement in the OR that makes the patient humane | - Advocating to re-enter humanness in the OR | Minor |
| **10** | Berlinger,  2016,  USA | Time-out: The Professional and Organizational Ethics of Speaking Up in the OR | Review article  8p | Summarizes key research and discusses the professional and organizational ethics of patient safety | - Address limitations and ethical dimensions of checklists in surgery  - Checklist fatigue add to safety problem  - Medical systems are explicitly hierarchical, must be countered for checklists to be  effective  - Safety as basic part of professional ethics  - Communication as an ethically significant activity | Minor  - Safety |
| **11** | Bernstein,  2016,  USA | Not the Last Word: Two Patients, Two Operating Rooms, One Surgeon—Does The Math Add Up? | Address  - Bernstein  - Langerman  - Mello  6p | Advocating for review of data to assess safety of concurrent operations. | - B: Questioning “superstar culture” in surgery vs. team-based approach  - L: Team-based approach may endanger the surgeon-patient bond of trust  - M: Rigorous research needed on outcomes and preferences | Minor  - Safety |
| **12** | Booij,  2008,  Netherlands | Teamwork and the legal and ethical responsibility of the anaesthetist | Review article  5p | Operating teams are increasing, and collaboration requires protocols and rules to ensure patient safety. | - Traditionally, anaesthetists and surgeons have run their jobs parallelly  - Interdisciplinary collaboration involves  - Working together with shared responsibility  - Effective communication  - Uniformity of professional standards  - The anaesthetist as autonomous clinician, accepting responsibility and role | Minor  - Teamwork |
| **13** | Boukis,  1986,  Greece | A forensic pathologist in the surgery room. Positive and negative issues | Personal narrative  3p | Demonstrating the medico-legal advantages of having a forensic pathologist documenting facts in the OR | - Focus on a possible tension of deontological issues as a stranger in the OR | Minor  - Little ethics |
| **14** | Brenna,  2021,  Canada | Imperfect by design: the problematic ethics of  surgical training | Review article  4p | How do we ethically validate the current training model for surgeons, in which trainees are often given operative duties that could likely be better handled by a staff physician? | - System of internal balances that mitigate discrepancy in surgical skill  - Tension between the best interests of the individual patient and the responsibility  of the profession to train new surgeons  - Deontological values of best patient care would exclude medical trainees  - The health care system is starkly utilitarian, maximising societal utility  - This deontological vs utilitarian conflict cannot be reconciled ethically  - The modern physician as mediator of the tensions between the two values | Substantial |
| **15** | Bruce,  2015,  USA | Emergent Ethics Consultation  Requested From the Operating Room | Case report  1p | An emergency case in the OR where a Jehovah’s Witness needs blood transplants to survive. | - Poor preparatory work lead up to an acute dilemma inside the OR:  - Because of the severity of the decision, the patient’s wishes are essential. But the  non- JW wife questions the patient’s written statement  - The surgeon separates the ethical issue, which he forwards to the clinical ethicist | Minor |
| **16** | Bujold,  1992,  USA | DNR orders in the operating room | Comment  < 1p | Response to Cohen 1991 | - The central obligation is to help the patient and relieve suffering  - This includes resuscitation if the prognosis is favourable | Minor |
| **17** | Caruso,  2002,  USA | Perioperative do not resuscitate orders: Caring for the dying in the operating room and intensive care unit | Comment  4p | How to relate to a request for surgery combined with a DNR order?  Response to Waisel 2002 on multidisciplinary policy for perioperative DNR orders | - *Conditional treatment* (a distinct level of care) is harder for health care workers to  morally accept  - Different interpretations of beneficence, nonmaleficence and autonomy  - Cardiopulmonary arrest  - Respiratory arrest  - Extraordinary measures | Minor  - Consent |
| **18** | Clark,  1994,  USA | Ethical dilemmas and decisions concerning the do-not-resuscitate patient undergoing anesthesia | Review article  4p | Presentation of the moral  dilemmas DNR orders pose for Nurse Anaesthetists, and aid to make decisions that are most “right” for the patient. | - DNR meaning is elusive and no standard treatment of DNR orders in patients  receiving anaesthesia. Anaesthetists thus face many dilemmas:  - Anaesthesia agents inherently alter the patient’s entire state of being  - Resuscitation must not violate the patient’s right to die with dignity  - The anaesthetist must adhere to legal policies  - The decision involves patient autonomy, the practitioner’s sense of “doing  good or right,” and achieving a good surgical outcome | Minor  - Nursing |
| **19** | Cohen,  1991,  USA | Do-not-resuscitate orders in the operating room | Guideline  4p | Authors propose a policy of “required consideration” to reexamine DNR orders before surgery | - Preexisting DNR orders may be unethical in the OR.  - 5 questions to consider before entering the OR:  3. Does DNR orders compromise patient’s objectives? (Success rate)  4. Why are OR personnel reluctant to retain DNR orders? (Responsibility) | Minor  - Outside OR |
| **20** | Cohen,  1992,  USA | DNR orders in the operating room | Comment  <1p | Response to comments from Franklin and Bujold (1992) | - Real difficulties: Is withholding resuscitation of a dying patient an avoidable death? Argue against absolute rules. | Minor |
| **21** | Couper,  1992,  USA | DNR in the OR | Letter  < 1p | Response to Walker 1991 | - Anaesthesia is complex and artificial. Often involves some form of resuscitation.  - Patients may be poorly prepared for surgery (because of DNR)  - If awake, many patients would have changed their mind  - Emotional toll on the OR team | Minor |
| **22** | Craig,  1996,  Canada | Do not resuscitate orders in the operating room | Review article  6p | Review of the policies and management of DNR orders in the OR, emphasis on Canadian issues.  Showing how absolute views (DNR/no DNR) are incorrect. | Historical development:  - First: DNR patients need surgery – suspend DNR  - Then: Avoid breach of autonomy – keep DNR  - New guidelines: Discuss DNR options with patient. HCP may withdraw | Minor  - Guidelines  - Consent |
| **23** | Craig,  1998,  Canada | Do not resuscitate orders –managing the dilemma | Review article  6p | Examine the basis for the dilemma of DNR orders in the OR. “Respect for persons” as guiding principle. | - Build on previous analysis (1996)  - “Iatrogenic events” as a confounding element  - Anaesthetic measures fully understood only by Anaesthetists | Minor  - Consent |
| **24** | Daly,  1991,  USA | ‘Do-not-resuscitate’ orders during anesthesia and surgery | Response  < 1p | Response to Troug (1991) | - Argue that Troug, with his concept of the “special boundary” of the OR,  overrides patient autonomy | Minor |
| **25** | Darrow,  2020,  USA | The Potential for Undue Patient Exposure during the Use of Telementoring Technology. | Quantitative article  7p | Quantify the risk for violation of privacy when video and audio recording during surgical procedures. | - Telementoring is an ethical addition because:  - Provides tailored feedback and reduces complication risk  - Dynamic and effective mode of remote teaching  - May improve teaching in the developed world  - Implementation needs to balance security and accessibility  - Result: 1,13 exposures/min (0,409/min in OR) without prevention measures | Minor  - Information security |
| **26** | Duty,  2012,  USA | Live surgical demonstrations: an old, but increasingly controversial practice | Quantitative article  5p | Survey among urologists about the educational value, safety, and ethics of live surgical demonstrations. | - Advantages (inside OR): expert surgeons, access to advanced equipment, educate  multiple HCP – expert commentary and unfolding of procedure  - Disadvantages (inside OR): increased infection risk, surgeon distraction, conflict of  interest  - Surgeon’s position morally accentuated: indication, distractions, stress | Substantial |
| **27** | Ewanchuk,  2006,  Canada | Ethics review: Perioperative do-not-resuscitate orders - Doing ‘nothing’ when ‘something’ can be done | Review article  4p | Consider the origins of the DNR order in anaesthesia and critical care practice and examine current opinions regarding patients presenting for surgery with DNR orders. | - Two reasons for reluctance to adhere to DNR in the OR (non-applicable):  - Concerns regarding the DNR order: anaesthesiologists and surgeons are not  patient’s primary physicians; how to interpret the order and consent?  - Difficulties related to the nature of the OR environment: Resuscitation is  a normal function of the anaesthetics, medical facts differ  - OR is a poor location for deaths; staff stressors, blocks OR, family removed,  responsibility on the surgeon | Substantial |
| **28** | Fagerdahl,  2022,  Sweden | Moral Distress Among Operating Room Personnel  During the COVID-19 Pandemic: A Qualitative  Study | Qualitative article  9p | Explore OR team members’ experiences during the COVID-19 pandemic | - Moral distress as starting point  - Interview with 20 OR team members by nurse  - OR ward described as comfort zone  - Extra stress load: feeling unsafe during surgery, correcting team members, not  wanting to act as police | Minor  - Little ethics |
| **29** | Fina,  1994,  USA | Ethical issues: a chance to say goodbye. | Case report  4p | Case where parents were denied access to the OR and their dying baby | - Establishes the “liaison nurse role”, information and support to family  - OR staff’s stress and feelings of responsibility  - Urge to spare parents from traumatic details  - New policy:  - OR team determines if the OR environment is suitable for parents  - The surgeon may or may not remain in the OR | Minor  - Little ethics |
| **30** | Franklin, 1992,  USA | DNR orders in the operating room | Response  < 1p | Response to Cohen (1991) | - Stress that mechanical ventilation/vasopressor agents can be stopped  postoperatively  - Patients will more likely consent to surgery  - Easier to initiate life support in the OR despite uncertainty | Minor |
| **31** | Gabrielli,  2021,  Chile | Audio and panoramic video recording in the  operating room: legal and ethical perspectives | Literature review  5p | Provide a better ethical and legal understanding of  the new challenges concerning VR in the OR.  GDPR (EU) vs. HIPAA (US) | - Ethics in VR in the OR: The act of recording  - Respect for autonomy; patient and OR team  - Hawthorne effect, affecting non-technical skills  - Not interfering with OR security  - Ethics of VR in the OR: consequences and responsibilities of VR  - Confidentiality in use of video  - Edit or erase video (ownership) | Minor  - Legal issues |
| **32** | Gallant,  2018,  USA | How Should Trainee Autonomy and Oversight Be Managed in the Setting of Overlapping Surgery? | Ethics case  7p | Case of one surgeon overseeing two operations by residents, one of which has a postoperative complication | - Main issue for the authors is informed consent, the patient should decide  - Overlapping vs. concurrent surgery. Latter conflicts with all 4 ethical principles  - Surgeon’s responsibility: “At no point in surgical residency training is there a no-  supervision phase” | Minor  - Outside OR |
| **33** | Gallant,  2022,  USA | Patient Perceptions of Audio and Video Recording in the Operating Room | Qualitative review  7p | Determine surgical patients’ perceptions of hypothetical continuous audio-video OR recording | - Benefits: objective record, medical education and quality improvement, patient  education and curiosity, respect and dignity, affecting performance  - Risks: privacy and identifiability, data use, sharing, and security, consent and  ownership, profit and cost, affecting performance | Minor  - Little ethics |
| **34** | Gavrus,  2015  Canada | Skill, Judgement and Conduct for the First Generation of Neurosurgeons,  1900–1930 | Historical review  18p | Describes how the relationship between surgical skill and professional judgement is reflected in broader medical history | - Skills performed in the presence of a select audience: the specialist society  - Function of societies: negotiate new surgical knowledge/techniques, create a  moral economy, enforce professional standards, articulate a common identity  - Surgical epistemology anchored in experimental physiology and experimentation  - Moral character – arbitrating between surgical skill and judgement. Linked to  gentlemanly culture and integrity. Not seeking money or fame  - Surgical spectators discouraged because the technique, not the patient in focus  - Ideal: self-control in the OR; emotions and impulses. Align their self to the group | Substantial |
| **35** | Hadjipavlou,  2013,  Greece | Wrong site surgery. The maze of potential errors | Editorial  2p | Medical errors as t as the failure of a system involving cognitive, affective, and procedural aspects. | - Different types of failures in surgery – propagating into errors  - Procedural vs. judgemental errors  - Skill-based, rule-based and knowledge-based behaviour  - Psychological anchoring – latent error  - “Swiss Cheese” model – system responsibility  - Handling error in the OR – medico-legal consensus of waking the patient to obtain  informed consent to correct the error. | Minor |
| **36** | Hartley,  2019,  Canada | Dead on the table: A theoretical expansion of the vicarious trauma that operating room clinicians experience when their patients die | Review article  10p | Discuss 3 forces that shape OR care experiences: biomedical values, normative death discourse, and socially (un)sanctioned grief. | - Vicarious traumatization: empathetic engagement  - Second victimization: participating in adverse events, culpability  - Cultural aspects of OR:  - Biomedical values, reductionistic: unprepared for death, repressing emotions  - Good death discourse unavailable: surgery gruesome, isolated from family  - Disenfranchised grief, polarization between professional and personal care  - Clinicians must be acknowledged for the role as agents, experiencing and  perpetuating the sources of vicarious trauma and second victimization | Minor  - Psychology  - Behaviour |
| **37** | Heit,  2014a,  USA | Surgical Proctoring for Gynecologic Surgery | Guidelines  4p | Educate physicians about surgical proctoring and provide guidance for establishing a proctoring  program in gynecologic surgery | - Surgical proctoring: monitor, regulate, oversee surgical privileging to ensure  quality and safety for patients  - No patient-doctor relationship. Represents institution.  - US case law: No obligation to intervene if complications or substandard care  - But: ethical duty to prevent harm/take remedial action.  - Good Samaritan law should provide immunity for intervention | Minor  - Legal issues |
| **38** | Heit,  2014b,  USA | Surgical Proctoring for  Gynecologic Surgery | Response  <1p | Response to Wertheim (2014) | - Distinction between legal responsibility and ethical decision of the proctor  - Good Samaritan laws and Hippocratic Oath guide toward intervention | Minor |
| **39** | Hua,  2008,  USA | Medical treatment of Jehovah’s witnesses | Ethics case  3p | Case of patient attempted suicide by shotgun, in the OR discovered that he is a Jehovah’s Witness | - Moral discussion of 4 options:  1. Give blood: Best medical practice, beneficence. NB: spiritually beneficence  2. Use other fluids: Weighting beneficence and autonomy  3. Find sources for patient preferences: Informed consent, validating autonomy  4. Do not give transfusion: Autonomy, right to choose  - Bottom line: Medically justified in action 1 – standard of care | Substantial  - Case |
| **40** | Humbyrd,  2013,  USA | Judgments about deservingness | Ethics case  3p | Case of gang member with gunshot wounds to one hand. During the lengthy surgery, it is questioned if the work is worthwhile. | - Principle of nonjudgmental regard: Meet medical needs, regardless of cause or  worth. Many afflictions linked to behaviour. We would want it for our loved ones.  - Principle of beneficence: Put the patients’ good before their own. Oath of serving  patients’ interests  - Professional responsibility: practice nonjudgmental regard  - Social factors only valid to individualize care | Minor  - Outside OR |
| **41** | Husted,  2000,  USA | When is a health care system not an ethical health care system? Suspending the do-not-resuscitate order in the operating room. | Address  7p | Demonstrate that the policy of automatically suspending DNR in the OR is fundamentally flawed | - Describes the health care system as a contractual arrangement  - “The only authority relevant to any bioethical decision is the patient”  - Claim that the practice of suspending DNR orders violate the nature of health care  systems and thus, violates patients’ rights | Minor  - Outside OR |
| **42** | Hyson,  2019,  USA | Surgical Overlap: An Ethical Approach to Empirical Ambiguity. | Review article  14p | Examine overlap surgery for the surgeon, the hospital, and the patient,  based on 4 ethical systems (consequentialism, deontology, principilism, virtue ethics) | - Surgeons:  - C: Best outcomes for all, train future surgeons, higher volume, career  advancement  - D: Patient autonomy and informed consent, distributive justice  - P: Weighting benefits to patients vs society, potential harm, informed consent  - V: The “good surgeon”, providing proper care, motivation vital  - Surgeons bear the responsibility for the entire operation  - Surgical overlap is a disclosure issue, best approach is shared decision-making | Minor  - Outside OR |
| **43** | Igoe,  1993,  USA | Ethics in the OR: DNR and patient autonomy | Review article  3p | Examines the new Patient Self-Determination Act (1991) in light of a clinical case, literature review and Ethics Committee discussion | - The Patient Self-Determination Act created a dilemma to patients with DNR  orders in the OR – not automatically suspended. The Act makes no exception for  surgery  - Anaesthesia promotes cardiac instability. “Tying the physician’s hands”  - Surgeons as captain of the ship  - Death as a “bad outcome” or peaceful end in the OR | Minor  - Outside OR |
| **44** | Jackson,  1999,  USA | Goals- and values-directed approach to informed consent in the “DNR” patient presenting for surgery: more demanding of the anesthesiologist? | Editorial  4p | Response to Truog (1999): The goal-directed approach introduces ambiguity and returns the decision-making to the physician. | - Overview of guidelines on DNR orders in the OR: Required consideration and  renegotiation. Practice obstacles (in OR):  - Blurred distinction between anaesthesia and resuscitation  - Resuscitation is more successful in the OR  - Reluctance to invite death into the OR  - Misunderstanding of legal issues  - Question if patient autonomy profits from returning decisions to physicians | Minor  - Consent |
| **45** | Jesudason,  2023,  UK | Surgery should be routinely videoed | Review article  5p | Argue that video recording of surgery is an ethical duty to protect | - Ethics gap in surgery: promoting surgeon flexibility and lack of oversight  - Video benefits:  - Surgery invisible to outsiders, rely on reputation  - Improves transparency to patients/consent  - Improving justice; objective evaluation, reduced bullying, more accessible care  - AI quality improvement  - Potential objections: irrelevance/misconceptions, liability, privacy, confidentiality,  cost, added surgeon stress, distributive injustice and climate effect, consent  - Surgeons/institutions’ duties to protect – duty to implement routine recordings | Substantial |
| **46** | Jones,  2013,  USA | Operating one-handed: Emergency treatment of  Jehovah’s Witnesses | Ethics case  3p | Case of Jehovah’s Witness with ruptured aneurism and acute need of transfusion | - The scenario is interpreted through the 4 principles and legal regulation  - Hinges on the validity of surrogate decision-making: “substituted judgement  standard”  - The second best is “best interest standard”, beneficence based | Minor  - Consent |
| **47** | Jones,  2014  USA | Defining, aligning, or declining do not resuscitate during surgery | Ethics case  2p | Case of multimorbid patient with a DNR order, who develops VT in the OR | - Cannot resuscitate because it violates patient autonomy  - Only option is to do nothing or obtain consent from surrogate | Minor |
| **48** | Jones,  2008,  USA | Operative simulcasts: Patient’s donations to surgeon’s educations | Ethics case  2p | Question of giving a real-time educational surgery broadcast session on a surgical association meeting | - Replace ethical primacy of the patient with ego needs of surgeon and profession  - Educational value is poorer than edited video  - Operation converted to spectacle, exciting and thrilling show  - The patient needs all attention | Minor |
| **49** | Jones,  2002,  USA | Complying with advance directives in the operating room | Ethics case  2p | Case of patient with hypotensive cardiac arrest in the OR after car accident, and the discovery of a living will that opposes CPR | - Argues that the surgeon must establish if the condition for the living will is  accurate for the present situation. | Minor |
| **50** | Joris,  2011,  Belgium | End of life care in the operating room for non-heart-beating donors: organization at the University Hospital of Liege | Practice guidelines  4p | Describe end of life care for NHBD in our institution, including  the problems addressed, the solution proposed, and the remaining issues | - Common protocol (in OR):  - OR personnel do not participate in the decision  - Sufficient time to mourn vs keeping warm ischemia as short as possible  - Measures to protect organ viability: from analgo-sedation to volatile  anaesthetic  - Definition of death and deciding when brain death occurs | Minor  - Guidelines  - Little ethics |
| **51** | Keffer,  1992,  USA | Do-not-resuscitate in the operating room: Moral obligations of anesthesiologists | Review article  5p | Present the moral argument for making a choice based on patient autonomy and explicate the terms that will aid in understanding the moral argument. | - Routinely suspend DNR orders - no different from any other order on the chart.  Ignoring medicine as a moral enterprise:  - The good sought in the encounter is that of the patient, not the physician  - Conflict in the perception of the patient’s good - refer to another physician  - The anaesthesiologist’s moral duty transcends the duty of professional  competency | Minor  - Outside OR |
| **52** | Knipe,  2013,  UK | Past, present, and future of ‘Do not attempt resuscitation’ orders in the perioperative period | Address  3p | Request a change in DNR guidelines for patients in need of surgery | - Reasons to suspend DNR order in the OR:  - Cardiac arrest caused by anaesthetic or surgical intervention  - Everything should be done for survival in the OR  - Higher success rates from CPR  - Overlap between anaesthetic interventions and components of CPR  - Advance directives often not applicable to OR environment  - Argues that patients should decide whether to continue DNR order or not | Minor  - Outside OR |
| **53** | Lam,  2021,  UK | The Ethical Digital Surgeon | Review article  3p | Explore the ethical and regulatory consequences of the digital transformation of the operating room | - Digital robotic systems hope to improve safety and clinical outcomes in the future  - Large amount of data – risk of misuse, question of ownership/payment, consent  - “Digital surgeons” – minor decisions will be scrutinized, risk of litigation  - Function of digital surgery system if patients refuse to share data  - Introduction of AI systems in the OR needs regulation  - Digital surgeons need AI literacy | Minor  - Legal issues  - Quality |
| **54** | Leavitt,  2015,  USA | Live surgical demonstrations: An endangered species | Address  4p | Argue that live demonstration is a very valuable teaching tool and should remain a part of surgical education. | - Surgery - not cooking; involves immediate decision-making, creativity, spontaneity  - Patient benefits: therapies they cannot afford, expert highly motivated surgeons  - Rapid spreading of new techniques - across work fields. Shows workflow in the OR  - Downsides (in OR): Patient safety, surgeon distraction, OR traffic, unfamiliar OR,  missing equipment, language barriers, jetlag, liability issues, questionable motives  - Surgeon qualities: consistent technical quality, do not blame others  - Patient safety political correctness - result in guidelines hampering live  demonstrations | Minor  - Little ethics |
| **55** | Leung-Tack,  2022,  UK | Senior surgeons as role models in the operating theatre: a thematic analysis through the lens of aristotelian ethics | Qualitative article  7p | Examine role model behaviours of senior surgeons through the lens of Aristotelian (virtue) ethics | - Interviews with 6 *anaesthetic* trainees  - Major findings:  - Team work: valuing others as experts and team players. Building trust  - Captain of the ship: dictate the room/plan, lack holistic care, surgical persona  - Strong foundations: creating positive work environment, flat hierarchy  - Overriding character flaws: pursuit of power, God complex, surgical persona  - Behaviour as manifestation of character- teaching surgeons to ‘be’ not to ‘do’ | Substantial  - Teamwork with focus on virtue ethics |
| **56** | Levin,  2017,  USA | Overlapping and Concurrent Surgery: A Professional and Ethical Analysis | Guidelines  6p | Examine professional, bioethical, pedagogical, and regulatory considerations of concurrent surgery. Provide practical guidelines | - Overlapping surgery: staggered start, sequential start, coinciding cases  - Professionalism. Guidelines (“critical portion”)  - Beneficence “requires physicians to ensure that their actions are consistent  with their patients’ values, needs, and agreed-upon treatment”. A primary focus  - Nonmaleficence: elevated risk of trainees  - Justice: distributive justice, parsimonious care  - Recommendations within OR (1 of 7): portions of the procedure in which the  attending surgeon was not present in the OR should be documented | Minor  - Outside OR |
| **57** | Liverneaux,  2019,  France | Should we ban Live Surgery? | Editorial  5p | Draw practitioner’s attention to the risks and excesses of Live Surgeries | - Surgeon exposed to undeniable psychological stress  - No proof of pedagogical value – should ban live surgery  - “The art of surgery must be learned by apprenticeship.”  - “The human relationship lies at the heart of our work!” | Minor  - Little ethics |
| **58** | Lonchyna,  1997,  USA | To resuscitate or not ... in the operating room: the need for hospital policies for surgeons regarding DNR orders | Review article  19p | Discuss 3 possible approaches of a DNR order in the OR: automatically suspend, strictly adhere or reconsider the DNR order for the surgery. Call for surgical team policies. | - The OR is the best place for the patient to be if there is a need for resuscitation  - Permissible, a duty of the surgeon to perform a life-saving emergency operation  - Iatrogenic complication, the surgeon has a fiduciary obligation to do all possible  - Training and work: surgeons/anaesthesiologists are aggressive interventionalists.  - Anaesthesia: deliberate depression, then resuscitation/stabilization of vital signs  - OR personnel may feel inactivity is directly responsible for a patient's death  Patients not sent to the OR to die: bad outcome  - Automatically suspend: paternalistic infringement on patients’ right. Strictly  adhere: contrary to OR teams obligations. Solution: Required reconsideration | Substantial |
| **59** | Marsden,  2019,  USA | Ethical Issues Encountered During the Medical Student Surgical Clerkship | Qualitative article  6p | Content analysis of students’ reflection on ethical issues they encounter during surgical clerkship | - Obligatory essay after lecture on surgical ethics. Analysed according to the 4  principles.  - Decision-making the most frequent category (28%)  - Issues in the OR in 9% of essays:  - Staff interaction, conversation in the OR, supervision of trainees, surgical  hubris, disclosure of trainee role, running multiple ORs | Minor  - Outside OR |
| **60** | Mavroudis,  2007,  USA | The influence of Plato, Aristotle, and the ancient Polis on a programme for congenital cardiac surgery: The Virtuous Partnership | Personal narrative  5p | Description of a well-functioning programme for congenital cardiac surgery with reference to Platos *polis* and Aristotelian virtues | - Several “captains of the ship”. “Check your ego at the door”  - Surgeons operate together, tackle uncertainty together, discussion of operative  methods  - Striving for high professional level and collegial relationships | Minor  - Little ethics |
| **61** | Mavroudis,  2012,  USA | Ethical considerations for post-cardiotomy extracorporeal membrane oxygenation | Review article  7p | Explore ethical dilemmas arising from application of post-cardiotomy extracorporeal membrane oxygenation | - Virtuous physicians: knowledge, application, practice  - Inverse relationship between knowledge and courage  - Can intervention ever be contraindicated in the OR (with growing need)?  - Tacit maxim: “Nobody dies in the OR”  - Elicits administrative duties and emotional experiences  - Easier to transfer a patient “technically alive” out of the OR  - Transferring the patient also transfers some responsibility for failure  - Technological imperative immoral: suffering, benefits, life quality  - Should use pre-set goals for expected recovery. Ethics relevant for indications | Substantial |
| **62** | McKenna,  2012,  Canada | Competency-based professionalism in anesthesiology: continuing professional development | Lecture  10p | Discuss the attitudes, skills, and behaviours that define professionalism as it relates to the practice of anaesthesiology | - Professionalism: skills, knowledge, integrity + moral understanding, social contract  1. Professional conduct: Appropriate inter professional behaviour with team  members. Practice according to established safety practices. Implement  prevention strategies  2. Ethical practice: Advocating for patient’s rights to privacy, dignity when under  anaesthesia. Prioritizing mother vs foetus in emergency obstetrical care. Abiding  by patient’s directives regarding use of blood products  3. Legal aspects: Participate in maintenance of competence programs; life-long  learning. Manage conflicts and difficult people in the perioperative setting  4. Physician health: risk of errors/complications from suboptimal physical condition,  recognize high-risk events, concerns associated with physician aging | Minor  - Outside OR |
| **63** | McWilliams,  1976,  USA | Divided responsibilities for operating room asepsis: The dilemma of technology | Address  2p | Rituals in the OR must be replaced by procedures based on principles of aseptic technique | - Concerns for asepsis and patient safety are multidisciplinary. Cooperative effort  - The surgeon and anaesthesiologist are surrounded by technologic advances  - Automation cannot replace humans. Must be curbed by moral and legal  constraints  - Aseptic technique (for patients) vs. ritualism (for HCPs)  - Asepsis rules need constant revision | Minor |
| **64** | Michel,  2008,  Belgium | Responsibility in the Operating Theatre: The guidelines are still controversial | Review article  6p | Demonstrate how surgeons are morally and legally responsible for all patient treatment in the OR | - Surgeons’ contractual responsibility for mistakes made by the OR team  - The only one contractually bound to the patient (by law and by patient trust)  - Team members substitute themselves to accomplish the surgeon’s obligation  - Controversial - anaesthesiologists are independent specialists  - Solution? Partial contract responsibility, stipulating the tasks of team members,  institutional litigation, tacit agreement  - Law is tangled with the ethical foundation of the patient-physician relationship | Minor  - Legal issues |
| **65** | Mohr,  1999,  Germany | Ethical problems in caring for organ donors: the perspectives of physicians and nurses involved | Review article  13p | Focus on the ICU and OR phase of organ donor management and organ procurement, with emphasis on ethical conflicts | - Organ donors represent disturbing deviations from the common rituals  - Organ retrieval surgery  - Visiting recovery team harvesting organs  - Discontinuing anaesthesia by turning off ventilator. Traumatic experience  - Change in the objective of care, from treating a dying person to caring for organs  - To maintain the organs will improve the life of a stranger in need of a transplant | Minor  - Outside OR |
| **66** | Muhammad,  2022,  Pakistan | Surgeons and ethical challenges in operating room | Review article  7p | Highlight ethical problems arising in the OR, and their solutions in light of literature and guidelines | - Patients’ privacy: autonomy, dignity, confidentiality.  - Patients’ safety: professionalism, teamwork, interruptions (DID), checklists  - Overlapping surgery: multidisciplinary procedures, efficiency, resident autonomy,  definitions of critical portions of surgery  - Live surgery broadcast: dissemination of knowledge, to be treated as research.  Protocol, IRB, patient’s advocate, performing surgeon as active part  - DNR code in the OR: individual decision without compromising institutional policy | Substantial |
| **67** | Nagral,  2004,  India | Live operative workshops: A critique | Address  2p | Demonstrate how live operative workshops raise serious ethical questions | - Case of liver resection with fatal postoperative bleeding  - Live operation workshops often organized by private sector  - Promote institution or equipment  - Expensive medical equipment/technology (not addressing patients’ needs)  - The operation as glamourous and dramatic performance  - No educational focus; discussions, corrections. Risk of oversimplification.  - Surgeon responsibility for equipment, standards, OR | Minor |
| **68** | O’Connor,  2016,  USA | Salespeople in the Surgical Suite: Relationships between Surgeons and  Medical Device Representatives | Qualitative article  18p | Raise ethical questions about surgeons’ reliance on device reps for surgical assistance and concerns regarding levels of competence among OR personnel | - Complex relation; personal, target the surgeon, auxiliary, extending knowledge  - Benefiting from each other  - Work on commission, incentive to get a surgeon to use something more expensive  - Presence of reps needed for education of new devices (“early adopters”)  - Lack of skillset among surgeons, should know the equipment they use  - Conflict of interest? Both want the surgery to go fast and uneventful. But the  surgeon serves the patient, and the sales rep serves the company | Minor  - Little ethics |
| **69** | Philip-Watson,  2014,  UK | Live surgery at conferences – Clinical benefits and ethical dilemmas | Review article  4p | Give an overview of Live Surgery Broadcasts and explore possible use of pre-recorded alternatives | - Autonomy: paternalistic relationship  - Beneficence: surgeons’ motivation, distractions  - Respect: risk of compromising patient dignity and confidentiality  - Recommend the presence of a urologist as patient’s advocate in the OR, reduce  unfamiliarity for surgeons. Consider pre-recorded surgery. | Minor  - Outside OR |
| **70** | Pinzur,  2017,  USA | Concurrent Surgery | Address  2p | Argue that overlapping surgery may abide to the responsibility to the next generation of surgeons and patients | - Ethical overlapping surgery- surgeon present for key parts, efficiency in patient  setup  - Residents must obtain necessary skills – under supervision | Minor |
| **71** | Prentice,  2021,  USA/Canada | Surgical Teamwork and the Pragmatic Ethics of the Outcome | Qualitative article  14p | Show how focus on the team makes visible collective relations of surgical teamwork, reinforce the surgery’s goals, and provide moral lessons to team members | - Relational glue that binds teams - opposes the idea of an extended surgeon body  - Agency emerges from routines, outcome as shared goal - improving routines is  ethical  - Examining team practices as ethical action  - Disciplined training and routinized activities can lead to desired outcomes  - Agency as it emerges from within rational-technical practices  - Surgery: ethical imperative of return to routine functioning. Returning to normal  order – often with acknowledgment of the moral danger the moment held | Substantial |
| **72** | Prigoff,  2016,  USA | Ethical Recommendations for Video Recording in the Operating Room | Clinical guidelines  2p | Discuss the ethical issues that intraoperative recording pose and provide recommendations for their use | - Confidentiality: discussions of other patients  - Privacy: private conversation of the OR team  - Litigation | Minor  - Outside OR |
| **73** | Rhodes,  1994,  USA | An Alternate Opinion: Do-Not-Resuscitate Orders in the Operating Room | Comment  2p | Comment on Rosner (1994) | - Physician’s duty of beneficence  - If resuscitation is an integral part of anaesthesia:  - Not a patient choice  - Incoherent wish  - Does not violate patient autonomy | Minor |
| **74** | Roberts,  2002,  USA | Do-not-resuscitate orders in the OR--do they work for the patient? | Review article  3p | Describe arguments and ethics of DNR orders in the OR | - Surgeons: DNR orders suspended, bring the patient back to the preoperative state  - Anaesthesiologists: patients’ right to participate. Middle-of-the road agreement  - Perioperative nurses: OR is no place to die. Should be surrounded by family | Minor  - Outside OR |
| **75** | Rosner,  1994,  USA | Do-not-resuscitate orders in the operating room | Report  5p | Educational report on the issue of DNR orders in the OR in the State of New York | - 59% of anaesthesiologists assume DNR order is suspended  - Most anaesthesiologist would not honour a DNR order  - Arguments for suspending DNR orders:  - Artificial distinction between anaesthesia and resuscitation  - Cardiac arrest potentially reversible  - The order may not be valid in these circumstances  - Patients’ real motive may be euthanasia | Minor  - Little ethics |
| **76** | Ryan,  2002,  USA | The chapel and the operating room: the struggle of Roman Catholic clergy, physicians, and believers with the dilemmas of obstetric surgery, 1800-1900. | Historical review  34p | Discuss the influence of religion and justice on  Catholic physicians’ decisions of how best to resolve childbirth emergencies. | - Before 1900: craniotomy or caesarean operation to resolve difficult childbirth  - Catholicism, epigenetics/physiology – value of the foetus’ life  - Removing the uterus to save the mother  - 1884: Vatican banned craniotomy. Caesarean increased survival rate. Asepsis  - Religious and ethical concern shaped decisions  - Different belief systems – each physician had to decide appropriate measures  - Reluctance to learn new techniques in the OR  - Collaboration of the Chapel and the OR – due to uncertainty | Minor  - Outside OR |
| **77** | Sade,  2008,  USA | Broadcast of Surgical Procedures as a Teaching Instrument in Cardiothoracic Surgery | Guidelines  5p | Review of arguments and proposing new guidelines on the subject | - Positive sides: Benefits to patients/public/surgeons. Educational. Emotional drama  - Negative sides:  - Distraction of the surgeon  - Increased infection risk  - Diverted focus when communicating with audience  - Time pressure; delays, improper indication  - Surgeon unfamiliar with site or equipment  - Violation of confidentiality  - Misuse of surgical procedures for entertainment  - Ethical deliberation: weighing benefits and harms – relevant data do not exist, we  must therefore, rely on principles from ethical codes | Minor  - Little ethics |
| **78** | Satava,  2002,  USA | Laparoscopic surgery, robots, and surgical simulation: Moral and ethical issues | Review article  9p | Define moral issues that new technologies might bring to the field of surgery | - Laparoscopic surgery: New skills requirement and learning curve. Loss of skillset  required for open approaches. Conflict of interest. Surgery performed before  clinical evidence  - New technology:  - Harm: robot gone wild, wrong programming, responsibility  - Benefit: higher quality, alert systems, assess performance  - New issues: system in conflict with surgeon, too complex to understand, AI,  dependence on systems, implants to improve performance, skilled surgeons  outdated  - Many issues without objective criteria; professionalism, communication | Minor  - Outside OR |
| **79** | Scarlet,  2017,  USA | Surgery in Shackles: What Are Surgeons' Obligations to Incarcerated Patients in the Operating Room? | Guidelines  8p | Discuss challenges for surgery on incarcerated persons and future recommendations | - Presence of correction officers in OR; challenge trust, privacy, intimidation  - Likelihood of escape during surgery is extremely low  - Revealing of patient vulnerabilities may lead to abuse and victimization  - Should adhere to prison policies for similar situations  - Are current policies justified when looking at empirical data? | Minor  - Little ethics |
| **80** | Scarrow,  2018,  USA | A medical-legal perspective on overlapping surgery | Review article  5p | Describe the current literature on overlapping surgery and the ethics that guide behaviour | - Ethical considerations:  - Perform more surgeries, better access to care  - More training of junior surgeons  - More efficient OR time  - Additional risk because of less supervision  - Less efficient for the anaesthesia team  - Surgeon’s loss of focus | Minor  - Outside OR |
| **81** | Scott,  2012,  USA | Palliative surgery in the do-not-resuscitate patient: ethics and practical suggestions for management | Practice guidelines  12p | Suggestions of how to handle the complexities of DNR orders in the OR | - Palliative surgery challenge: patients’ negative right to refuse treatment vs.  physicians’ prerogative to refuse treatments inconsistent with standards of care  - Medical vs. moral objections. Evolve around patient’s life goals  - Focus not only on analgesia, but on dyspnoea, cough, sedation, anxiety, nausea | Minor  - Outside OR |
| **82** | Shapiro,  2019,  USA | Perioperative Advance Directives: Do Not Resuscitate in the Operating Room | Review article  7p | Describe factors that contribute to DNR orders in the OR, and current policy | - Outcomes of CPR – better in the OR  - Automatically suspension violates patient autonomy. “Required consideration”  - Procedure-directed and goal-directed interventions | Minor  - Outside OR |
| **83** | Shapshay,  2016,  USA | The ethics of running multiple operating rooms simultaneously: Is this Ghost surgery? | Editorial  2p | Assert the unethical consequences of concurrent surgery | - The breach of a sacred contract with the patient  - Potentially increased complication rate  - Potentially poorer surgical outcome  - Failure to educate trainees | Minor  - Consent |
| **84** | Smith,  1994,  USA | DNR in the OR | Guidelines  8p | Review the ethical aspects and guidelines of DNR orders in surgical patients. Policy suggestion. | - The patient bears the risks, analogy to Jehovah’s Witness  - Beneficence; patient knows best (life goals). Strongest argument; doctors’ duty.  - Prognosis of CPR better in OR, in line with patient goals  - Difficult to give proper anaesthesia  - Doctors feel responsible for deaths in OR. DNR violates nature of profession, harm  - OR deaths may have devastating effect on doctors  - Solution: cause of cardiac arrest central (disease or iatrogenic), doctor’s discretion | Minor  - Outside OR |
| **85** | Smith,  2013,  USA | Surgeon responsibility | Editorial  2p | Discuss the responsibility of deciding when the surgeon has adequate skills for a procedure | - “The moral, professional and even legal obligations of a surgeon are profound and  ideally supersede any financial or personal considerations”  - “A patient entrusting his or her quality of life and very survival to a surgeon  creates an ethical bond that has few parallels in society”  - “It is the surgeon’s conscience that should be the primary determinant of whether  he or she is qualified to perform a particular operation” | Minor |
| **86** | Smith,  2000,  USA | Do-not-resuscitate orders in the operating room: required reconsideration | Review article  4p | Defend the policy of "required reconsideration" as a more flexible and ethical protocol then alternatives | - Special considerations in the OR:  - Consent to surgery is not implicit consent to suspension of DNR procedure  - Anaesthesia manipulates body and may lead to resuscitation  - Cardiac arrest might be result of therapeutic intervention  - Fine balance, the practitioners must be able to use all skills  - Parallel to Jehovah’s Witness  - “DNR decisions are moral decisions, are based on the patient's own value system,  and belong solely to the patient. They are not medical decisions.” | Minor  - Outside OR |
| **87** | Steil,  2019,  Germany | Robotic Systems in Operating Theaters: New Forms of Team-Machine Interaction in Health Care | Congress  12p | Discuss the challenges of new team–machine interactions and their  consequences for Health Information Systems, exemplified by robotic systems in operating theatres | - Surgical viewpoint: influence self-conception. Responsibility, safety, managing  complications, defining quality  - Robotics viewpoint: difficult assigning responsibility. Hybrid action, shared  autonomy. Skill evaluation, norm setting, skill deprivation  - Information systems viewpoint: non-living entities; sensors/access to patient data  - Legal viewpoint: new systems, risks not fully known, no specific standards,  liability/responsibility, hard-to-entangle collaboration, “human in the loop”,  sensitive data  - Ethical viewpoint: Cooperative partners – moral status, hierarchic role. Supremacy  of human autonomy, control over the machine when self-learning algorithms are  non- transparent. Empirical and theoretical questions evolving | Substantial |
| **88** | Taylor,  2020,  USA | Ethical and medicolegal aspects in the management of neurosurgical emergencies among Jehovah's Witnesses: Clinical implications and review | Case report  5p | Present the case of a Jehovah’s Witness in need of an urgent hemi-craniectomy, which was delayed due to missing documentation regarding the use of blood products | - Operative delay  - JW’s have lesser blood loss. Death in OR due to blood loss extremely rare  - The procedure had a low risk of large haemorrhage  - Ethical considerations: the 4 principles  - Delay caused more damage  - In line with DNR order – which surrogates may consent to  - Practical solutions; synthetic agents, preoperative techniques, precautions to  reduce blood loss | Minor  - Outside OR |
| **89** | Tchana-Sato,  2019,  Belgium | Successful clinical transplantation of hearts donated after circulatory death using normothermic regional perfusion | Case report  6p | Present two cases of successful heart transplantation after donors’ circulatory death and ante-mortem interventions | - Ante-mortem intubation, sedation, cannulation, heparinization  - Shorten warm ischemic time, improving graft outcome  - Expose donor to risk, modifies end-of-life care  - Restoration of systemic circulation and mechanical cardiac functioning  - Invalidating previous declaration of death?  - Death must persist long enough for the brain to die  - Success measured in function of recipient | Minor  - Little ethics |
| **90** | Toledo-Pereyra,  2009,  USA | Gentleman surgeon | Editorial note  3p | Defining the gentleman surgeon as demonstrating caring, respect and help | - Care: improving surgery, showing compassion toward healing the whole human  - Respect: better communication, positive environment  - Help: engages in clinical practice, teaching and research | Minor  - Little ethics |
| **91** | Trafford,  1978,  USA | Crisis in the operating room | News article  4p | Describing rising challenges related to the profession of surgery in US | - Questions of motivation; income, unnecessary procedures  - Salesmen present and acting in the OR  - Residents perform operations without the patient’s knowledge  - Non-certified surgeons  - Variation in surgical care (institutional level) | Minor  - Little ethics |
| **92** | Truog,  1991,  USA | Do-not-resuscitate" orders during anesthesia and surgery | Review article  3p | Review of the practice regarding DNR orders in preoperative and postoperative care | - Iatrogenic cause is not valid to suspend DNR order (occurs also outside OR)  - Arguments supporting suspension of DNR order:  - Artificial separation between anaesthesia and resuscitation (implied)  - Every arrest is potentially reversible  - Need to suspend DNR order to have the full benefit of anaesthesia  - Must define temporal limitation of suspension  - Address how to deal with arrests that do not respond to initial resuscitation | Minor  - little ethics |
| **93** | Truog,  1992,  USA | DNR in the OR | Comment  <1p | Comment on Walker (1991) | - Resuscitation inside OR  - Higher success rate. No pain.  - Anaesthesiologists may give better care  - Should be the presumed  - Confusion on anaest. practice, definition of arrest. Need involvement of anaesth. | Minor |
| **94** | Truog,  1999,  USA | DNR in the OR: a goal-directed approach | Review article  7p | Provide a framework for guiding negotiations between patient and clinicians, and suggest options for managing DNR orders in surgery | - Advocate for a goal-directed approach  - Patients worry about subjective, personal issues, not technical ones  - Anaesthesiologists will not have their “hands tied”  - Ambiguity is no more than for surgery at large  - Procedure-directed approach necessary when lack of trust | Minor  - Little ethics |
| **95** | Tungpalan,  2001,  USA | DNR orders in the OR | Review article  4p | Review guidelines for DNR orders in the OR in relation to a hypothetical case | - Effectiveness of CPR increases in the OR  - Quickly discovered  - Easily reversible  - Iatrogenic cardiac arrest – more likely to be overridden  - Fear of malpractice litigation  - Feelings of guilt/responsibility  - Invalid - not a situation considered by the patient | Minor  - Little ethics |
| **96** | Ueda,  2013,  Japan | Perspectives: Professionalism and cardio-thoracic surgery | Review article  5p | Address principles and virtues for medical professionalism, especially among surgeons. Pointing at the role of professional societies. | - 3 fundamental principles: patient welfare, patient autonomy, social justice  - 10 commitments, 3 important for cardiothoracic surgery:  - Professional competence; maintaining knowledge and skills  - Improving quality of care; safety, use of resources, optimize outcomes  - Professional responsibilities; education, communication, internal assessment  - In the OR: trust. Non-Technical Skills (NOTSS): Situational awareness, decision  making, communication, teamwork, leadership | Minor  - Outside OR |
| **97** | Vanderpool,  2009,  USA | Surgeons as mirrors of common life: A novel inquiry into the ethics of surgery | Congress  2p | Analysis of special dimensions of ethics of surgery | - Ethics of surgery reflects the common values upon which societies rest  - Like ordinary citizens, surgeons are called upon to display virtues of character and  to manifest actions predicated on respect for human dignity and autonomy  - Ethically neglected: rescue surgery  - Powerful effective interventions, invasions into human body  - Patients surrender, emotions of fear or gratitude | Minor |
| **98** | VanNorman,  2005,  USA | Ethical issues of importance to anesthesiologists regarding organ donation after cardiac death | Review article  5p | Discuss ethical and legal issues for anaesthesiologists involved in DCD | - Two ethically conflicting events; altruism and care of the dying patient  1) Withdrawal of life-sustaining interventions: suffering, dyspnoea, anxiety  2) Care of dying patient: cultural, spiritual needs of patient and family. Ensure  enough time to declare death.  3) Donation of vital organs; preserving organ function, anticoagulants may hasten  death. Minimise time after death – viability uncertain  - Some anaesthesiologists are competent in end-of life care  - Involvement of anaesthesiologists give the impression that the donor is not dead | Minor  - Only address anaesthesiologists |
| **99** | VanNorman,  2003a,  USA | Ethical issues and the role of anesthesiologists in non-heart-beating organ donation | Review article  5p | Discuss ethical challenges in DCD and the anaesthesiologist role | - Doing harm; invasive monitoring devices, transferral to OR, family denied  presence. Added suffering by medication (preventing euthanasia) or hasten death  - Physician conflict of interest, saving organ viability. Professional image harmed  - Patient dignity  - “Slippery-slope” issues; definition of irreversible arrest  - Anaesthesiologists not required, as donation does not require sedation | Minor  - Only address anaesthesiologists |
| **100** | VanNorman,  2003b,  USA | Another matter of life and death: What every anesthesiologist should know about the ethical, legal, and policy implications of the non-heart-beating cadaver organ donor | Review article  11p | Discuss ethical and legal issues for anaesthesiologists involved in NHBCD | - Moral support for NHBCD: autonomy, beneficence  - Against NHBCD: nonmaleficence; deprivation of life, suffering, compromised care,  violating doctor-patient relationship trust, loss of dignity, harm professional image  - Logical Slipp.Slope: acceptance of withdrawing life support – leads to euthanasia  - Pragmatic SS: social, economic, or psychological pressures to accept the slope  - Timing of death, Irreversible cardiac arrest, Candidates for NHBCD  - The presence of an anaesthesiologist is unnecessary and potentially harmful.  Argues for a specialist in end-of-life care | Minor  - Only address anaesthesiologists |
| **101** | Waldman,  2018,  USA | The art of being in 2 rooms at 1 time: Ethical issues with overlapping surgery | Review article  3p | Review of ethical issues of overlapping surgery in dermatology with case scenario | - “Critical portion of procedure”: diversity of operations - difficult defining critical  portion, many dermatologic procedures not complex, nor present high risk  - No common definition of critical portion: allow for individual assessment of  residents’ skills. But power imbalance makes residents or nurses unable to object  - Patients are awake and aware of who’s operating – but still in power imbalance  - Overlapping surgery in dermatology is a benign practice | Minor  - Little ethics |
| **102** | Walker,  1991,  USA | DNR in the OR. Resuscitation as an operative risk | Review article  6p | Argue for the permissibility of honouring intraoperative DNR orders, addressing physicians’ moral concerns | - Physician’s interest:  - Death in OR = bad outcome. Investigations, suspicions  - Threatens surgeon’s command in the OR. Lessens authority  - Doctors’ sense of responsibility. OR set up to minimise risks/complications,  allow for preventable deaths untenable. The only *moral* reason. Moral agents  - Analogous to Jehovah’s Witness – want surgery but limits the help they accept  - Double effect – like morphine for palliative patients. BUT: cannot reverse the bad  effect because of patient’s right to refusal  - DNR orders distinguished from assisted suicide - because of doctors’ intent to help | Substantial |
| **103** | Walker,  1992,  USA | DNR in the OR | Response  <1p | Response to Couper, Franklin and Truog (1992) | - Definitions of resuscitation, distinguishing from prearrest interventions | Minor |
| **104** | Wertheim,  2014,  USA | Surgical proctoring for gynecologic surgery | Comment  1p | Comment on Heit “Surgical proctoring for gynecologic surgery”, opposing that proctors should not be allowed to intervene in proctored surgery | - Hippocratic oath, do the right thing: if anyone in OR needs help, you help them  - Denying proctors to intervene counters the reason to have them; patient safety | Minor |
| **105** | Willassen,  2015,  Norway/ Sweden | Student nurses' experiences of undignified caring in perioperative practice - Part II | Qualitative article  12p | Written stories from OR nurse students on what they perceived and interpreted as undignified caring in perioperative practice | - Unprofessional acts:  1) Render patient invisible; not greeting, not properly introducing, busy preparing  2) Ignore patient’s worry/pain; not listening, ignoring body language, ignoring pain  3) Treating the patient as an object; ignoring existential meaning, not explaining  - Humiliating behaviour:  1) Speaking derisively; commenting body, fat shaming – HP humiliated  2) Blaming patient; unhealthy lifestyle, preparations, bodily posture  - Discussion: Unfamiliar to culture, sensible to unethical practice. Moral distress,  inner ethical conflict in witnessing abuse. Lack of moral courage | Substantial |
| **106** | Youngner,  1985,  USA | Psychosocial and ethical implications of organ retrieval | Guidelines  4p | Address the disturbing effects organ-retrieval might have on personnel in ICU and OR | - OR personnel rarely exposed to dead people, death as defeat  - After surgery: ventilator turned off, coarse closing of the body, sent to morgue  - The objective of surgery is removal of tissue and someone else’s welfare  - 3 moral problems:  - Donor’s welfare is not the rationale behind surgery  - Organ-retrieval process seems to violate respect for persons as ends  - Disrespect for the dead | Substantial |
| **107** | Youngner,  1991,  USA | DNR in the operating room. Not really a paradox | Editorial  2p | Comment on Walker (1991) | - Doctors’ sense of responsibility  - Maintaining homeostasis – core of professional practice, moral agency  - Resuscitation – the only part doctors could forego  - Ambiguities will always remain | Minor |
| **108** | Yun,  2022,  South Korea | Tele-consent using mixed reality glasses (NREAL) in pediatric inguinal herniorrhaphy: a preliminary study | Quantitative article  9p | Evaluate usefulness of tele-consent using mixed reality technology for caregivers and surgeons during paediatric  inguinal herniorrhaphy | - Increased understanding for caregivers (direct views from OR)  - No delay in consent  - Less effort for surgeon  - Enhance legitimacy for surgeon’s decision  - Circumvent restricted access to hospitals/ORs (like Covid) | Minor  - little ethics |
