# Supplementary material for: Ethics in the operating room: a systematic review
Source: BMC Med Ethics. 2024 Nov 9;25:128. doi: 10.1186/s12910-024-01128-7 (PMC11550563; doi:10.1186/s12910-024-01128-7)
Supplement: Supplementary file 3 — Supplementary Material 3. [file 12910_2024_1128_MOESM3_ESM.pdf]

Flowchart of searches in Medline and Embase

Illustration of number of hits in Medline (blue) and Embase (green)

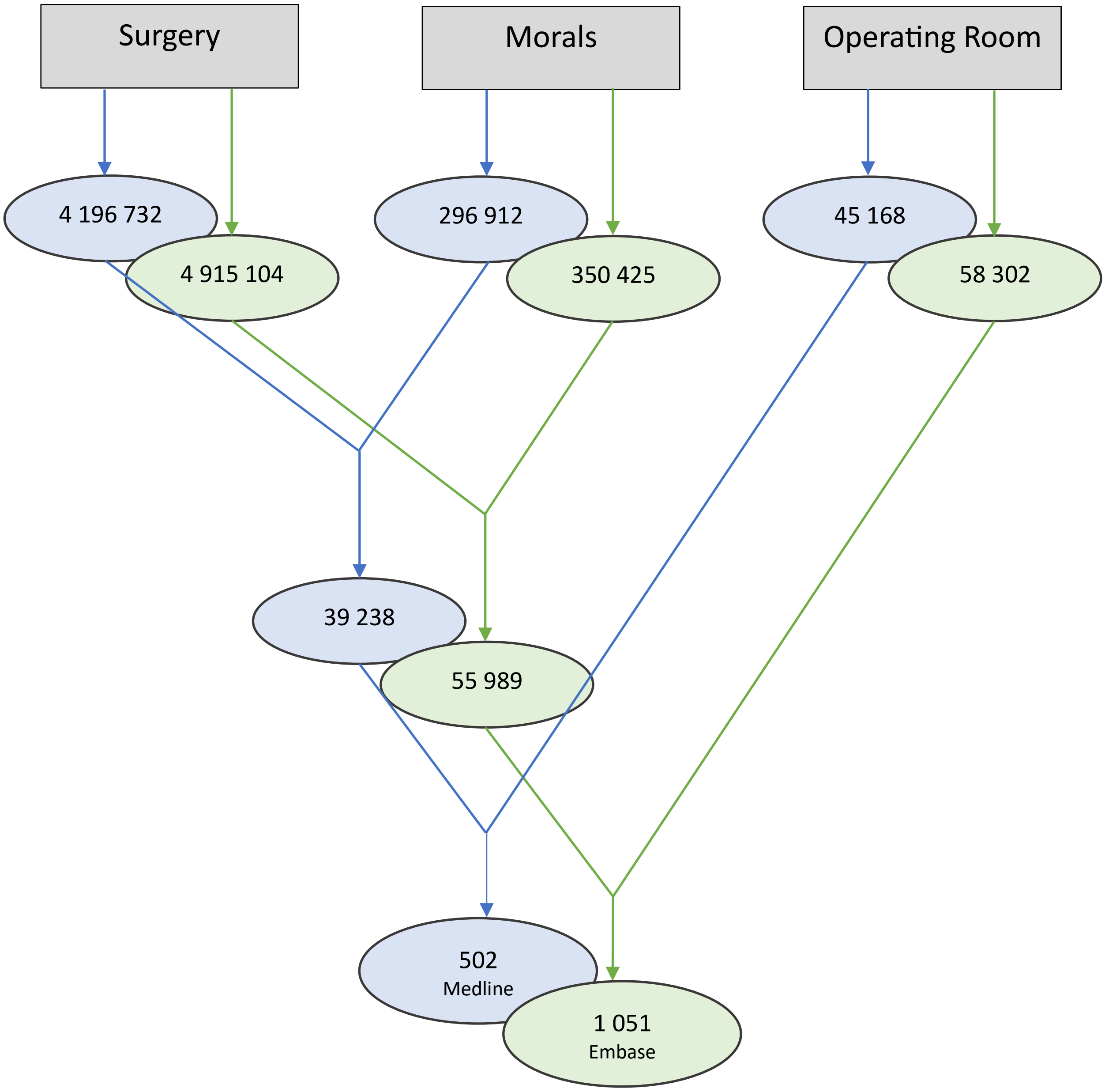

In Medline, of all publications on Surgery and Ethics (39 238), 1,28% publications include the Operating Room (502). In Embase, of all publications on Surgery and Ethics (55 989), 1,88% publications include the Operating Room (1 051).

This search was conducted 13th of April 2024, and is an approximation of the original search. The hits are based on a search limit of year (2022) with an addition of days in 2023 (40 days) multiplied by the average hits per day in 2023.

Medline: search history 13th April 2024

Search History (28)

# ▲	Searches	Results	Type
1	exp Specialties, Surgical/ or exp Surgeons/ or exp Surgical Procedures, Operative/ or exp Postoperative Complications/ or exp Surgical Equipment/ or exp Orthopedic Procedures/	4055265	Advanced
2	(surger* or surgic* or surgeon* or surg* complication* or operatively* or operative* treatment* or operative* management* or operative* technique* or operative* procedure* or operative* time* or operative* field* or preoperative* or pre-operative* or postoperative* or post-operative* or perioperative* or peri-operative* or intraoperative* or intra-operative* or reoperation* or re-operation*).ab,ti,kw.	2797994	Advanced
3	(operation* or operative*).kw.	6882	Advanced
4	1 or 2 or 3	5282858	Advanced
5	limit 4 to (danish or english or norwegian or swedish)	4423550	Advanced
6	exp Morals/ or exp Ethics/	185635	Advanced
7	(moral* or ethic*).ab,ti,kw.	204829	Advanced
8	ethics.fs.	77086	Advanced
9	6 or 7 or 8	342513	Advanced
10	limit 9 to (danish or english or norwegian or swedish)	314796	Advanced
11	exp Operating Rooms/	16200	Advanced
12	(operat* room* or surg* room* or operat* theat* or surg* theat* or surg* facilit*).ab,ti,kw.	46199	Advanced
13	11 or 12	52880	Advanced
14	limit 13 to (danish or english or norwegian or swedish)	48353	Advanced
15	5 and 10	41584	Advanced
16	10 and 14	713	Advanced
17	15 and 16	562	Advanced
18	5 and 14	38194	Advanced
19	limit 5 to yr="1900 - 2022"	4154160	Advanced
20	limit 10 to yr="1900 - 2022"	293502	Advanced
21	limit 14 to yr="1900 - 2022"	44565	Advanced
22	19 and 20	38804	Advanced
23	21 and 22	493	Advanced
24	limit 5 to yr="2022 - 2023"	388468	Advanced
25	limit 10 to yr="2022 - 2023"	31115	Advanced
26	limit 14 to yr="2022 - 2023"	5500	Advanced
27	24 and 25	3963	Advanced
28	26 and 27	86	Advanced

Embase: search history 13th April 2024

Search History (33)

# ▲	Searches	Results
1	exp Surgery/ or exp Surgeon/ or exp Postoperative Complication/ or exp Surgical Equipment/	6846085
2	(surger* or surgic* or surgeon* or surg* complication* or operatively* or operative* treatment* or operative* management* or operative* technique* or operative* procedure* or operative* time* or operative* field* or preoperative* or pre-operative* or postoperative* or post-operative* or perioperative* or peri-operative* or intraoperative* or intra-operative* or reoperation* or re-operation*).ab,ti,kw.	3996638
3	(operation* or operative*).kw.	13284
4	1 or 2 or 3	7757356
5	limit 4 to (danish or english or norwegian or swedish)	6729844
6	limit 5 to conference abstract	1504001
7	5 not 6	5225843
8	exp Morality/ or exp Ethics/	397572
9	(moral* or ethic*).ab,ti,kw.	296409
10	8 or 9	540974
11	limit 10 to (danish or english or norwegian or swedish)	501182
12	limit 11 to conference abstract	125403
13	11 not 12	375779
14	exp Operating Room/	57163
15	(operat* room* or surg* room* or operat* theat* or surg* theat* or surg* facilit*).ab,ti,kw.	68389
16	14 or 15	88096
17	limit 16 to (danish or english or norwegian or swedish)	81841
18	limit 17 to conference abstract	18510
19	17 not 18	63331
20	7 and 13	61348
21	13 and 19	1443
22	20 and 21	1213
23	7 and 19	51661
24	limit 7 to yr="1902 - 2022"	4855315
25	limit 13 to yr="1902 - 2022"	345522
26	limit 19 to yr="1902 - 2022"	57299
27	24 and 25	54969
28	26 and 27	1021
29	limit 7 to yr="2022 - 2023"	545574
30	limit 13 to yr="2022 - 2023"	44736
31	limit 19 to yr="2022 - 2023"	9149
32	29 and 30	9309
33	31 and 32	274
